# HiTIMED: hierarchical tumor immune microenvironment epigenetic deconvolution for accurate cell type resolution in the tumor microenvironment using tumor-type-specific DNA methylation data

**DOI:** 10.1186/s12967-022-03736-6

**Published:** 2022-11-08

**Authors:** Ze Zhang, John K. Wiencke, Karl T. Kelsey, Devin C. Koestler, Brock C. Christensen, Lucas A. Salas

**Affiliations:** 1 Department of Epidemiology, Geisel School of Medicine, Dartmouth College, Lebanon, NH USA; 2 Department of Neurological Surgery, University of California San Francisco, San Francisco, CA USA; 3 Institute for Human Genetics, University of California San Francisco, San Francisco, CA USA; 4 Departments of Epidemiology and Pathology and Laboratory Medicine, Brown University, Providence, RI USA; 5 Department of Biostatistics & Data Science, Medical Center, University of Kansas, Kansas City, KS USA; 6 Department of Molecular and Systems Biology, Geisel School of Medicine, Dartmouth College, Lebanon, NH USA; 7 Department of Community and Family Medicine, Geisel School of Medicine, Dartmouth College, Lebanon, NH USA

**Keywords:** DNA methylation, Deconvolution, Tumor microenvironment, Epigenetics, Cancer, Immune microenvironment, Tumor angiogenesis

## Abstract

**Background:**

Cellular compositions of solid tumor microenvironments are heterogeneous, varying across patients and tumor types. High-resolution profiling of the tumor microenvironment cell composition is crucial to understanding its biological and clinical implications. Previously, tumor microenvironment gene expression and DNA methylation-based deconvolution approaches have been shown to deconvolve major cell types. However, existing methods lack accuracy and specificity to tumor type and include limited identification of individual cell types.

**Results:**

We employed a novel tumor-type-specific hierarchical model using DNA methylation data to deconvolve the tumor microenvironment with high resolution, accuracy, and specificity. The deconvolution algorithm is named *HiTIMED*. Seventeen cell types from three major tumor microenvironment components can be profiled (tumor, immune, angiogenic) by *HiTIMED*, and it provides tumor-type-specific models for twenty carcinoma types. We demonstrate the prognostic significance of cell types that other tumor microenvironment deconvolution methods do not capture.

**Conclusion:**

We developed *HiTIMED*, a DNA methylation-based algorithm, to estimate cell proportions in the tumor microenvironment with high resolution and accuracy. *HiTIMED* deconvolution is amenable to archival biospecimens providing high-resolution profiles enabling to study of clinical and biological implications of variation and composition of the tumor microenvironment.

**Supplementary Information:**

The online version contains supplementary material available at 10.1186/s12967-022-03736-6.

## Background

Beyond clonally-derived tumor cells, abundant and heterogenous cells that harbor these tumor cells constitute the tumor microenvironment (TME) [1]. The TME plays an essential role in tumor differentiation, growth, and invasion [2]. The TME comprises a spectrum of cell types responsible for immune and angiogenic responses [2]. When antitumor immune responses are triggered, inflammatory cells populate the TME, including natural killer (NK) cells, active cytotoxic CD8 T cells, memory CD4 T cells, pro-inflammatory macrophages, and dendritic cells (DC). In contrast, a TME that contributes to functional evasion of tumor immune response includes Foxp3 + regulatory T cells (Tregs), exhausted CD8 T cells, inactive macrophages, and myeloid-derived suppressor cells (MDSCs) [1]. Non-tumor stromal cells and endothelial cells remodel the angiogenic microenvironment to support tumor growth and invasion [3]. Also, the plasticity of epithelial cells plays a critical role in tumor progression [4]. The dynamic interactions between tumor cells and other cells in their microenvironment can promote tumor progression [3].

Tumor immune subtypes can be identified based on immunological gene expression profiling [5]. Tumors that are highly characterized by pro-inflammatory cytokines and T cell infiltration, i.e., immunologically hot tumors, have a better response rate to immune checkpoint inhibitors compared to immunologically cold tumors, which have a relatively low level of immune cell infiltration [6]. However, the binary classification of hot and cold tumors oversimplifies the broader underlying immune landscape in TME. In the angiogenic microenvironment, tumors that are inclined to promote endothelial cell proliferation by producing vascular endothelial growth factor (VEGF) to develop new blood vessels can be targeted by angiogenesis inhibitors [7], e.g., cancers of the lung, kidney, breast, colon, and rectum [8]. Thus, understanding the heterogeneity of TME can guide therapy response and prognosis [1].

Gene expression and DNA methylation have been used to estimate cell composition in complex mixtures and include both reference-based and reference-free methods. CIBERSORT is a prominent reference-based method developed for deconvolving immune cell types using mRNA expression data [9]. The accuracy of cell composition estimates using gene expression approaches is limited by variability in cell-specific gene expression across cells and the feature-space of gene expression data. DNA methylation is an epigenetic modification associated with gene regulation and is essential to lineage specification in development to establish and preserve cellular identity [10]. There are three notable advantages to reference-based DNA methylation methods compared with RNA-based approaches in estimating cell composition. First, DNA is more stable than RNA. Second, the covalent addition of a methyl group to a cytosine is binary, tracking with cell count. Third, using standard measurement approaches, the feature space to define reference profiles of cell-specific DNA methylation is at least 40-fold that of the typical gene expression feature space and can be up to 2000-fold higher [11]. We have established and created extended libraries for reference-based DNA methylation deconvolution that result in improved accuracy and performance for peripheral blood immune cell deconvolution [12, 13]. Tissue-specific reference-based libraries have also been developed to infer cell-type composition in the brain, breast, and skin [14, 15].

Initial approaches to deconvolve the TME using DNA methylation have been described. *MethylCIBERSORT* and *MethylResolver* have succeeded in resolving 10 and 12 cell types, respectively [16, 17]. However, due to the complexity and heterogeneity of the cell types in the TME, existing methods lack accuracy, specificity, and detailed cell types. Both the *MethylCIBERSORT* and *MethylResolver* methods used data from cancer cell lines rather than data from primary cancer cells. This is potentially problematic for deconvolution as cancer cell lines harbor additional epigenetic alterations as compared to primary tumors. [18]. Also, instead of using organ-specific epithelial cell type DNA methylation signatures, *MethylResolver* used a universal standard reference for tumor purity estimation in all tumor types.

To address the limitations of existing methods and to enhance the accuracy and utility of TME deconvolution, we developed a novel DNA methylation-based algorithm that employs a tumor-type-specific hierarchical model and broadens the number of immune cell types that are deconvolved. Our method, called Hierarchical Tumor Immune Microenvironment Deconvolution (*HiTIMED),* uses deconvolution libraries specific to tumor type, identifying the most cell-discriminatory CpG sites for each cell type in each tumor type context, resulting in 12 libraries per tumor type. Our method also organizes deconvolution into the three major tumor microenvironment components (tumor, angiogenic, immune), resulting in the ability to resolve a total of 17 cell types in the TME: tumor, epithelial, endothelial, stromal, basophil, eosinophil, neutrophil, monocyte, dendritic cell (DC), B naïve (Bnv), B memory (Bmem), CD4T naïve (CD4nv), CD4T memory (CD4mem), CD8T naïve (CD8nv), CD8T memory (CD8mem), T regulatory (Treg), and natural killer (NK) cells, in 20 carcinoma types. *HiTIMED*'s ability to resolve tumor cellular composition with high resolution promises a better understanding of cell heterogeneity in the TME and offers new opportunities to study more complex relationships of the TME with etiologic exposures, patient outcomes, and response to treatment.

## Results

### *HiTIMED* tumor-type-specific hierarchical model, library development, and cell projection

**Fig. 1:**
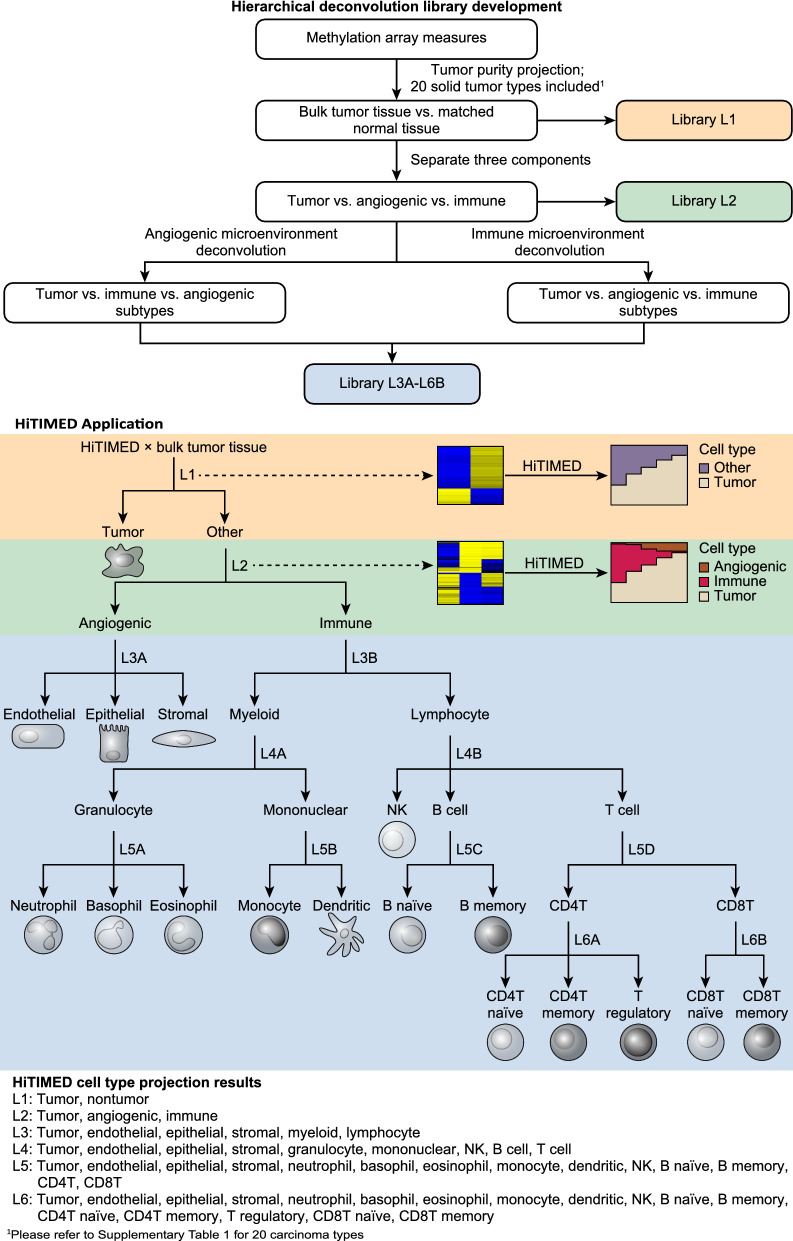
Structure of the *HiTIMED* tumor-type-specific hierarchical model, library development, and cell projection. For each carcinoma type, 12 libraries in 6 hierarchical layers (Library L1 – Library L6B) were optimized to estimate cell proportions. The first layer uses a tumor-type-specific reference library to deconvolve the tumor cell fraction from other cell types (Library L1). The second layer uses a library to separate tumor, angiogenic, and immune components (Library L2). Similarly, the third to the six layers use libraries to deconvolve angiogenic and immune cell subtypes (Library L3A-L6B)

*HiTIMED* employs a novel tumor-type-specific hierarchical model to deconvolve the TME. To develop *HiTIMED* we used discovery data from 6726 samples across 20 types of carcinomas and matched normal or normal-adjacent tissue. In addition, 26 samples for three angiogenic/non-immune cell types, and 61 samples for 13 immune cell types were included (Additional file 1: Table S1). Twelve libraries in 6 hierarchical layers were optimized for each carcinoma type to estimate cell proportions. The first layer (Library L1) uses a tumor-type-specific reference library to deconvolve the tumor cell fraction from other cell types (Fig. 1). Library L1 was developed by identifying the top 1000 most informative differentially methylated CpG sites from cancer-normal comparisons using the *InfiniumPurify* pipeline [19]. To discern tumor, immune, and angiogenic cells Library L2 and subsequent libraries were developed using the *Meffil* package [20], which used *limma* linear regression with empirical Bayes adjustment statistics to reduce methylation profiles to top 100 cell-type-specific hyper- and hypo-methylated CpGs. Then, two reference libraries in layer 3 of the hierarchical deconvolution were applied. Library L3A discerns the angiogenic microenvironment and deconvolves endothelial, epithelial, and stromal cell components. Library L3B separates lymphoid and myeloid cell fractions in the immune microenvironment. In the fourth layer, Library L4A distinguishes granulocytes and mononuclear cells under the myeloid lineage, and Library L4B separates NK, B, and T cells, in the lymphocyte lineage. In the fifth layer, Library L5A discerns neutrophils, basophils, and eosinophils, under the granulocyte lineage, and Library L5B discriminates monocyte and dendritic cells under the mononuclear cell lineage. Library L5C differentiates B naïve and B memory cells under the B cell lineage, and Library L5D was developed to detect CD4T and CD8T cells under the T cell lineage. In the sixth layer, Library L6A recognizes CD4T naïve, CD4T memory, and T regulatory cells under the CD4T lineage, and Library L6B differentiates CD8T naïve and CD8T memory under the CD8T lineage.

Cell proportions in the tumor TME were projected hierarchically using the above-mentioned Libraries. In the first layer, tumor and nontumor proportions were predicted by the probability density of methylation levels of Library L1 CpGs using the *InfiniumPurify* pipeline. From the second layer to the sixth layer, Libraries L2 to L6B were used in conjunction with the constrained projection quadratic programming approach described by Houseman et al. [21] to project the proportions of angiogenic and immune cells in the nontumor component from the first layer hierarchically by weighting the lower layer cell projections to the higher layer cell projections. In this manner, we identified 20 sets of 12 Libraries—one for each type of carcinoma—to optimally deconvolve the tumor microenvironment. The *HiTIMED deconvolution* function in the *HiTIMED* package was created to deconvolve the TMEs with a user-specified tumor site and layer. The package is available on https://github.com/SalasLab/HiTIMED.

### *HiTIMED* validation

To validate tumor purity estimates from *HiTIMED*, we compared the *HiTIMED* projected tumor cell proportion with the existing tumor purity estimation methods on publicly available tumor data. *InfiniumPurify* is a methylation-based and validated method for tumor purity prediction [19]. *HiTIMED* projected tumor proportions correlate significantly with the *InfiniumPurify* predicted tumor purities across tumor types (Additional file 2: Figure S1). Although highly correlated for most tumor types, five tumor types demonstrated correlation coefficient less than 0.5 (cholangiocarcinoma, kidney papillary, pancreatic, stomach, and thyroid carcinoma). To further validate our method for those five tumor types, we showed that *HiTIMED* tumor specific library has a clearer methylation distinction between tumor and normal samples compared to the *InfiniumPurify*’s default library for tumor purity estimation (Additional file 2: Figure S2). Furthermore, among thyroid carcinomas, we observed a cluster of tumors with lower tumor cell proportions from *HiTIMED* compared with *InfiniumPurify*. The heatmap demonstrated a more similar methylation state of the clustered tumors with controls compared to other tumors, which was not captured by *InfiniumPurify* (Additional file 2: Figure S3A). Follow-up uncovered that the cluster is predominantly composed of non-invasive follicular thyroid neoplasm with papillary-like nuclear features [22], and non-invasive follicular thyroid tumor purity is significantly lower than the invasive papillary thyroid carcinoma (Additional file 2: Figure S3B). Several tumor purity estimation methods, including those that use data sources other than DNA methylation, were compared to *HiTIMED*. These included methylation-based *MethylCIBERSORT* [17]*, MethylResolver* [16]*, LUMP* [23]*,* gene expression-based *ESTIMATE* [24]*,* somatic copy-number-based *ABSOLUTE* [25]*,* image stain-based immunohistochemistry *IHC* [26]*,* and consensus measurement of purity estimations (CPE) [26]. The results demonstrated significantly and highly correlated tumor cell projections with *HiTIMED* as compared to other established methods (Additional file 2: Figure S4). To validate the immune cell projections from *HiTIMED,* we deconvolved 12 immune cell artificial mixture samples whose ground truth immune composition across 12 cell types was known (Additional file 1: Table S2). All 12 immune cells showed a highly significant correlation between *HiTIMED* prediction and ground truth and low RMSE. 8 out of 12 cell types showed Pearson's correlation coefficients (R) over 0.90, and 11 out of 12 cell types showed R over 0.80 (Additional file 2: Figure S5). Although the scatterplots demonstrated slight under-prediction for some CD4T cell subsets and slight over-prediction for some CD8T cell subsets, the *HiTIMED* prediction for total T cells was highly accurate (R = 0.98, RMSE = 1.38, Additional file 2: Figure S6).

To validate *HiTIMED* in angiogenic microenvironment projection, we identified publicly available purified epithelial [27] and endothelial cells [28] for *HiTIMED* deconvolution (Additional file 1: Table S2). In the normal human intestinal epithelium, *HiTIMED* predicted on average 78.7% epithelial cell (SD = 6.3, Additional file 2: Figure S6). In human vein endothelial cells, *HiTIMED* predicted on average 87.6% endothelial cells (SD = 3.6, Additional file 2: Figure S7).

### *HiTIMED* deconvolution performance compared to the existing methods

To demonstrate the advantages of using *HiTIMED* to deconvolve tumor microenvironment, we compared its performance with *MethylCIBERSORT* and *MethylResolver. HiTIMED* encompassed all cells that can be captured by *MethylCIBERSORT* and *MethylResolver* except for macrophage, and offered 8 additional unique cell types (Additional file 2: Figure S8A). When comparing the performance of *HiTIMED*, *MethylCIBERSORT*, and *MethylResolver* on the 12 immune cell artificial mixture samples for the cell types that can be estimated by all three methods, HiTIMED showed the best performance with the mean absolute error 3.54% (SD = 3.3) compared to *MethylCIBERSORT* (Mean = 3.64%, SD = 2.4) and MethylResolver (Mean = 15.2%, SD = 16.7) (Additional file 2: Figure S8B).

### *HiTIMED* deconvolution of twenty types of carcinoma

**Fig. 2:**
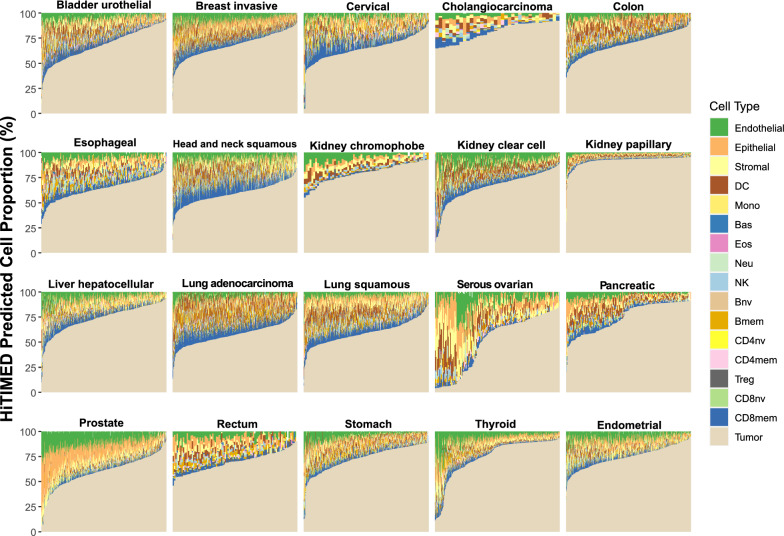
Cell composition differs substantially and captures sample heterogeneity using *HiTIMED* projected proportions. Seventeen cell types were captured for each sample by tumor type

To further investigate the utility of *HiTIMED*, we identified variation in TME cell proportions among 5986 carcinoma samples from 20 tumor types using DNA methylation data from multiple sources, including TCGA and GEO. The HiTIMED projected cell proportions for each tumor are illustrated in stacked bar plots (Fig. 2) and boxplots (Additional file 2: Figure S9). Due to the limited sample size for the TCGA ovarian cancer data set, additional publicly available samples were pooled (Additional file 1: Table S2). We assessed the variation in the immune component of the TME for all tumors, and the within-tumor variation across patients in the immune component was highest in lung adenocarcinoma, muscle-invasive bladder carcinoma, kidney clear cell carcinoma, head and neck squamous cell carcinoma and cervical carcinoma (Additional file 2: Figure S10A). Assessing variation in the tumor angiogenic microenvironment uncovered the highest within-tumor variation across patients in prostate, thyroid, stomach, pancreatic, and cervical carcinomas (Additional file 2: Figure S10B). The results implied potential high variability in immune- and angiogenic- related treatment response in those tumors.

**Fig. 3:**
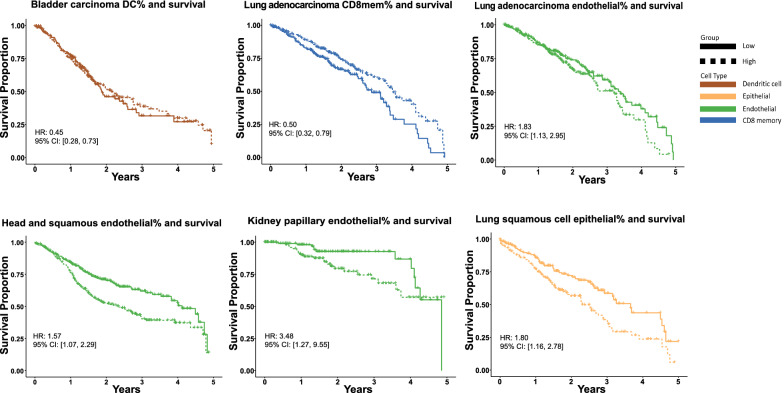
Tumor microenvironment heterogeneity measured by *HiTIMED* impacts 5-year survival in cancer patients. Kaplan–Meier survival curves with statistically significant hazard ratios from Cox proportion hazard models with age, gender, tumor stage, tumor proportion, and other cell-type proportions adjusted by comparing survival in higher than median value (High) to lower than or equal to median group (Low) for B memory, CD8T memory, dendritic cell, Tregs, epithelial, endothelial, and stromal cells in pan-cancer survival analyses

The association of specific cell type prevalence in TME with cancer patient survival is a major area of interest [29]. The high resolution of *HiTIMED* enables us to study cell-type prevalence and survival without potential confounding by other cell types. We investigated the relationship of seven quantitatively prominent and clinically relevant immune and angiogenic cell types in TME with patients' 5-year survival. We tested the association of *HiTIMED*-projected Treg, Bmem, DC, CD8mem, epithelial, endothelial, and stromal cells, respectively, with survival using Cox proportional hazard models adjusted for age, gender, tumor stage, *HiTIMED*-projected tumor proportion, and other cell-type proportions (Treg, Bmem, DC, CD8mem, epithelial, endothelial, stromal) by tumor type. Patients were stratified on the median value for each cell type. Statistically significant hazard ratios (HR) are demonstrated in Additional file 1: Table S3. We observed worse 5-year survival outcomes with higher than median level endothelial cell proportions in lung adenocarcinoma (HR 1.83, 95% CI [1.13, 2.95]), head and neck squamous cell carcinoma (HR 1.57, 95% CI [1.07,2.29]), and kidney papillary carcinoma (HR: 3.48, 95% CI [1.27, 9.55]) (Fig. 3). In lung squamous cell carcinoma, a higher than median level epithelial cell proportion is associated with a worse 5-year survival outcome (HR 1.80, 95% CI [1.16, 2.78]) (Fig. 3). For immune cells, better 5-year survival outcomes were observed for higher than median level DC and CD8mem proportions in bladder carcinoma (HR: 0.45, 95% CI [0.28, 0.73]) and lung adenocarcinoma (HR: 0.50, 95% CI [0.32, 0.79]) (Fig. 3). We compared two Cox models in kidney clear cell renal cell carcinoma with and without adjustment for cell types for a sensitivity analysis. We observed a higher effect estimate for the association of stromal cell prevalence and survival, a smaller effect estimate for the similar association of Treg prevalence and survival, and the association of the estimated DC prevalence with survival turned from significant to insignificant with survival after controlling for additional cell types (Additional file 2: Figure S11). This clearly suggests that adjusting for cell types in survival analysis is crucial for both understanding the nature of these cellular interactions and interpreting their association with patient outcomes. Additional Kaplan-Meier survival curves for the significant cell proportion associations adjusting for age, gender, and tumor proportion with survival are shown in Additional file 2: Figure S12.

**Fig. 4:**
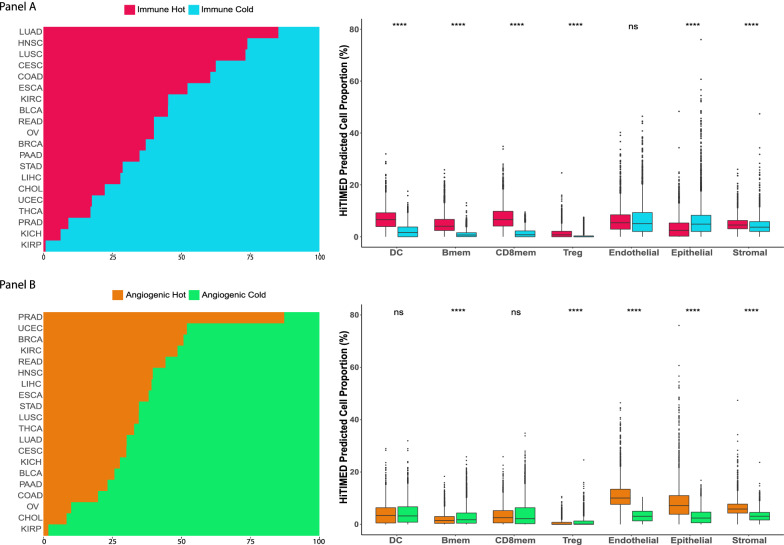
Immune/angiogenic hot and cold tumors are distinguished using *HiTIMED*-based PAM clustering. Panel **A**. Immune hot and cold subtype proportions by TCGA tumor type and comparisons of major *HiTIMED*-projected cells between immune hot and cold tumors. Panel **B**. Angiogenic hot and cold subtype proportions by TCGA tumor type and comparisons of major *HiTIMED*-projected cells between angiogenic hot and cold tumors. Please refer to the Abbreviations section for acronyms

**Fig. 5:**
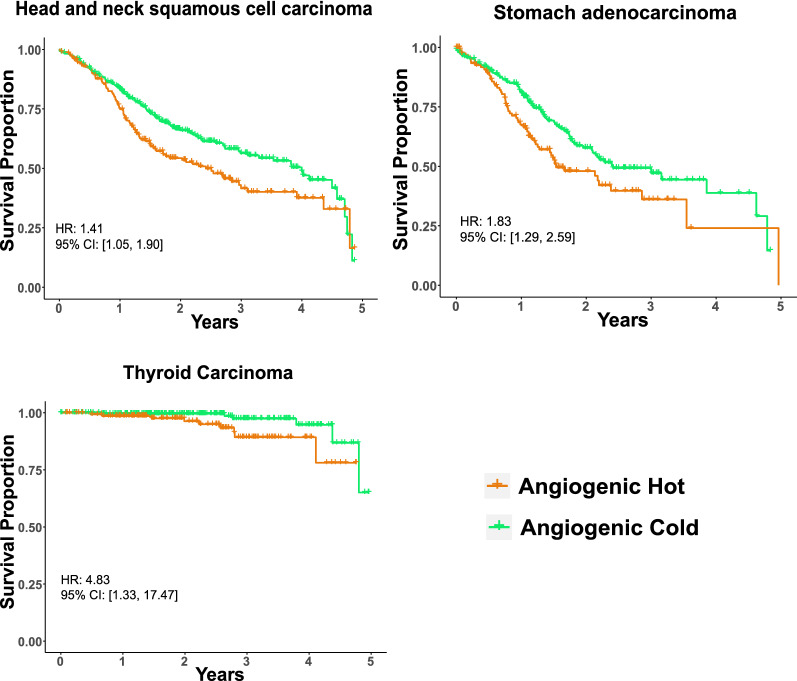
Angiogenic hot and cold tumors impact 5-year survival curves in head and neck squamous cell carcinoma, thyroid carcinoma, and stomach carcinoma. Hazard ratios are from Cox models adjusting for age, gender, and tumor stage

**Fig. 6:**
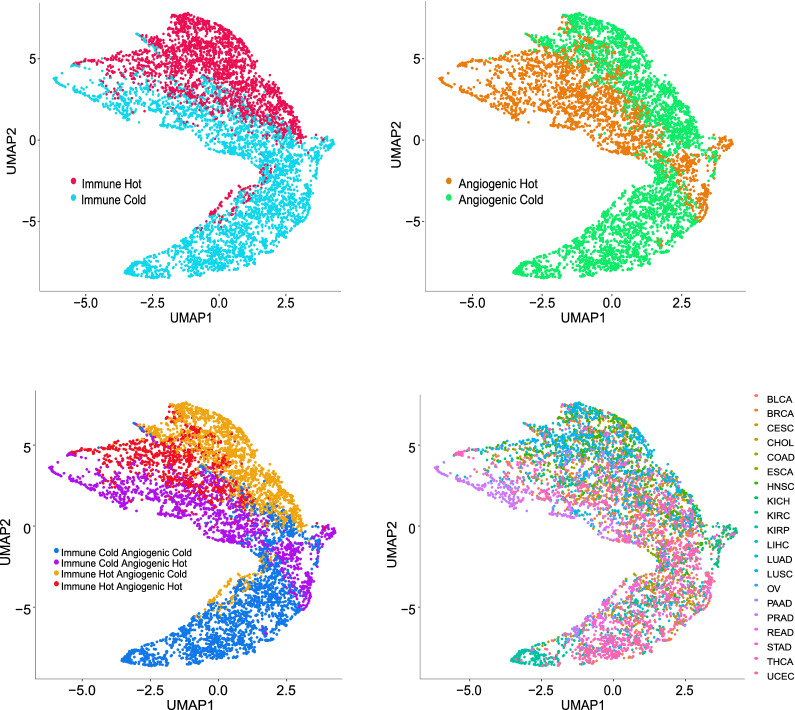
Independently of the tumor type TCGA samples can be classified by *HiTIMED* immune hot and cold subtypes, angiogenic hot and cold subtypes, and immune and angiogenic hot and cold subtypes. Uniform Manifold Approximation and Projection for Dimension Reduction (UMAP) clustering was used to classify the samples based on the *HiTIMED* TME cell composition, colored by tumor type and the angiogenic/immune classification. Please refer to the Abbreviations section for acronyms

Cell profiling in TME can be used to identify tumor immune subtypes [30]. Previous research used consensus partition around medoids (PAM) clustering to classify head and neck cancer immune hot and cold tumors based on predicted tumor cell fractions [17]. Similarly, based on the *HiTIMED*-projected immune microenvironment compositions, the TCGA carcinomas were classified as immune hot or cold by higher or lower immune proportion in two PAM clusters (Fig. 4A). In the immune hot tumors, we observed significantly higher proportions of dendritic cells (Δ = 3.28%, p-value = 8.5e-271), B memory cells (Δ = 3.39%, p-value < 2.2e-308, CD8 memory cells (Δ = 5.42%, p-value < 2.2e-308), and T regulatory cells (Δ = 0.87%, p-value = 3.4e-92), compared to immune cold tumors after adjusting for age, gender, and tumor type (Fig. 4A). We also used the consensus PAM clustering to classify the TCGA carcinomas as angiogenic hot or cold based on the *HiTIMED*-projected angiogenic microenvironment compositions (Fig. 4B). In the angiogenic hot tumors, we observed significantly higher proportions of endothelial cells (Δ = 7.29%, p-value < 2.2e-308), epithelial cells (Δ = 4.12%, p-value = 1.3e-221), and stromal cells (Δ = 2.97%, p-value < 2.2e-308) adjusting for age, gender, and tumor type (Fig. 5B). Cox proportional hazard models were applied to interrogate the 5-year survival difference between immune/angiogenic hot and cold tumors, adjusted for age, gender, and tumor stage (Fig. 4B). Worse 5-year survival outcomes were observed for angiogenic hot tumors in the head and neck squamous cell carcinoma (HR 1.41, 95% CI [1.05, 1.90]), stomach adenocarcinoma (HR: 1.83, 95% CI [1.29, 2.59]), and thyroid carcinoma (HR 4.83, 95% CI [1.33, 17.47]) (Fig. 5). Four groups of tumor clusters were generated by combining the immune and angiogenic hot and cold classification (Additional file 2: Figure S13A). Significantly differential survival outcomes were observed in clear cell renal cell carcinoma, thyroid carcinoma, stomach carcinoma, and cervical carcinoma across four clusters (Additional file 2: Figure S13B). The UMAPs demonstrated explicit tumor clustering by immune and angiogenic hot and cold subtypes (Fig. 6).

According to recent immunogenomic landscape analyses that leveraged multi-component genome-scale data sets, TCGA tumors were classified into six major immune subtypes, i.e., C1: wound healing, C2: IFN-γ dominant, C3: inflammatory, C4: lymphocyte depleted, C5: immunologically quiet, C6: TGF-β dominant [30]. *HiTIMED* deconvolution showed the lowest levels of immune cells in the C4: lymphocyte depleted and C5: immunologically quiet tumors and the highest levels of immune cells in C2: IFN-γ dominant and C6: TGF-β dominant. (Additional file 2: Figure S14A). A Higher resolution deconvolution with *HiTIMED* revealed a significantly higher DC proportion (p-value = 1.81e-08) and lower CD8mem proportion in C6 TGF-β dominant compared to C2 IFN-γ dominant tumors (p-value = 0.016, Additional file 2: Figure S14B).

### Cell-independent tumor DNA methylation alterations with HiTIMED cell projection in colon cancer

**Fig. 7:**
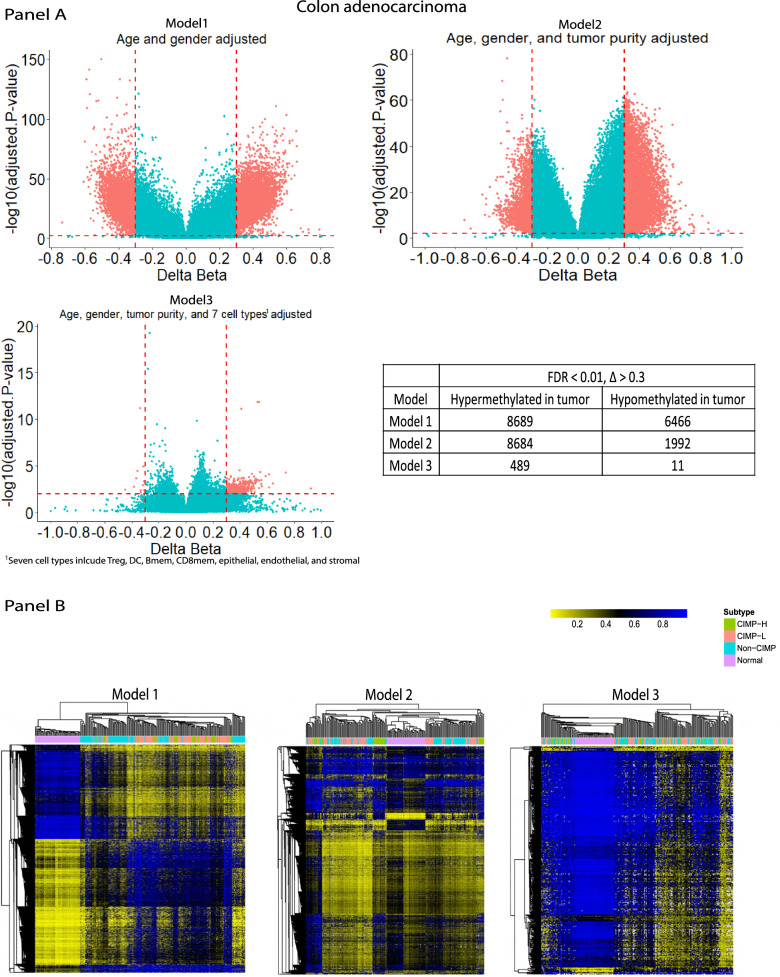
EWAS output comparisons across three models. Panel **A**. Model 1 adjusted for age and gender. Model 2 adjusted for age, gender, and *HiTIMED-*projected tumor purity. Model 3 adjusted for age, gender, *HiTIMED-*projected tumor purity, DC, CD8mem, Bmem, Treg, epithelial, endothelial, and stromal cell proportions. Delta betas larger than 0.3 and FDR smaller than 0.01 were used as the cut-off for statistically significant DMC identification. Panel **B**. Heatmap with Manhattan distance clustering and colon cancer CIMP subtypes colored were generated per model

Epigenome-wide association studies (EWAS) have been widely employed on cancer to identify altered methylation patterns between cancerous and normal tissues [31–34]. However, with the lack of high-resolution profiling of cell composition, current studies were incapable of identifying cell type independent methylation alteration in cancer. Now with *HiTIMED*, we investigated how a complete adjustment for TME cell composition impacts the identification of DNA methylation alterations in tumors compared with normal adjacent tissue. We tested models comparing methylation profiles between colon adenocarcinoma and adjacent-normal samples with adjustment for age and gender and with or without adjusting for *HiTIMED-*projected cell proportions. Adjusting for age, gender, and eight of the most prevalent cell types resulted in a dramatic attenuation of identified CpGs with significant differential methylation in tumor versus normal tissue (Δ > 0.3, FDR < 0.01) (Fig. 7A). Interestingly, the cell-type independent differentially methylated CpGs (DMCs) appeared to be more agnostic to the colon cancer CIMP subtypes than the DMCs identified from the unadjusted models (Fig. 7B). These results provide clear utility for isolating tumor-specific DNA methylation alterations, which has implications for basic cancer biology and developing treatment strategies.

### *HiTIMED* deconvolution and treatment response

To investigate how the TME is associated with treatment response, we applied *HiTIMED* to two publicly available data sets. One includes first-line chemotherapy drug-sensitive and -resistant metastatic colorectal cancers (mCRC). The other contains triple-negative breast cancer (TNBC) patients with and without recurrence in chemotherapy-treated and nonchemotherapy-treated arms after locoregional therapy (Additional file 1: Table S2). In mCRC, we observed significant lower levels of dendritic cell (Δ = 2.26%, p-value = 0.02), NK cell (Δ = 1.19%, p-value = 0.04), basophil (Δ = 0.53%, p-value = 0.01), neutrophil (Δ = 1.25%, p-value = 0.03), and a significantly higher tumor proportion (Δ = 7.74%, p-value = 0.03), in FOLFOX or FOLFIRI drug-sensitive patients compared to drug-resistant patients (Additional file 2: Figure S15). In TNBC, significantly lower levels of B memory cells and CD8T memory cells were observed in relapsing tumors in both the chemotherapy treatment arm (Bmem: Δ = 0.99%, p-value = 0.04; CD8mem: Δ = 2.18%, p-value = 0.04) and the nonchemotherapy treatment arm (Bmem: Δ = 1.92%, p-value = 0.004; CD8mem: Δ = 2.64%, p-value = 0.01) (Additional file 2: Figure S16).

## Discussion

Previous gene expression and DNA methylation-based deconvolution approaches for TME cell composition have had some success for major cell types [16, 17, 35]. However, due to the across-tumor-type diversity and within-tumor-type heterogeneity of the TME, substantial gaps still exist in tumor type specificity, cell projection accuracy, and cell-type resolution for TME deconvolution. Here, we present *HiTIMED*, optimized to more accurately, specifically, and exhaustively deconvolve the TME. *HiTIMED* has three major advantages compared to the existing algorithms: high cell-type resolution, tumor-specific libraries, and cell-projection accuracy optimization. Firstly, *HiTIMED* provides high-resolution profiling of the cell types in TMEs. Seventeen cell types in total among 3 TME components (tumor, immune, angiogenic) are projected by *HiTIMED*. In the immune microenvironment, closely related lymphocyte subtypes, including subtypes of CD4T and CD8T cells, and granulocyte subtypes are captured by *HiTIMED*. In the angiogenic/non-immune microenvironment, epithelial, endothelial, and stromal cells are profiled by *HiTIMED* separately as their roles in TME could be functionally very different. Furthermore, numerous variables from *HiTIMED* predicted cell types offer more opportunities to study the associations between TMEs and clinically relevant outcomes. For instance, studies have demonstrated CD8mem to Treg ratio as an indicator of the immune balance between cytotoxic and regulatory immunity, corresponding to the immunotherapy response [36–38]. Also, DC to NK ratio was studied in a mouse colon cancer model to enhance the antitumor effect as DC plays a crucial role in NK cell activation [39]. The high resolution of *HiTIMED* projection provides novel opportunities to exploit the cellular composition of the TME to discern patient prognosis and response to therapy. Although it can be argued that single-cell RNA sequencing technologies can offer a similar resolution of cell profiling in TME, DNA methylation-based deconvolution is immensely more cost-effective, less laborious, and is amenable to archival biospecimens where cells are no longer intact. Secondly, *HiTIMED* uses DNA methylation signatures that are specific to tumor type. Most of the existing methods developed a universal reference library for all types of tumors [16, 40]. Although, it is possible to estimate tumor purity with a signature that captures generalizable DNA methylation changes across all tumor types. The use of tumor-specific DNA methylation signatures maximizes the power of detecting most differentially methylated CpGs as tumors are genetically and epigenetically very different by tumor type. Although one algorithm has developed multiple libraries based on tumor type, cell lines were used rather than primary tumors [17]. Studies have shown consistently differential DNA methylation profiles between cancer cell lines and primary tumor samples [18, 41]. Finally, *HiTIMED* optimizes cell projection accuracy by employing a novel hierarchical model for deconvolution. With the high resolution of cell mixture deconvolution, bias can be generated with inevitable noise for cells under similar or the same lineage. The hierarchical model enhances the projection of the primary cell types in the specific lineage niche in a stepwise manner. For example, Library L3A in *HiTIMED* was designed to target angiogenic microenvironment deconvolution. As a result, the library collapsed all immune cells into one group but separated epithelial, endothelial, and stromal cells for optimal discernment. Although tumor purity and major immune cells were validated for accuracy in the previously existing methods, unlike *HiTIMED*, extensive deconvolution of immune cell types has not been validated in other methods [16, 17].

Understanding the TME with a standardized and cost-effective approach enables precision medicine. Studies have demonstrated TME's association with chemo- and immunotherapy responses and prognosis [1, 42, 43]. The balance between cytotoxic and regulatory immunity dictates tumor behavior in the immune microenvironment [36]. When the balance favors cytotoxic immunity, tumor elimination is promoted. On the contrary, tumor escape is facilitated when the balance tips toward regulatory immunity. CD8T cells are one of the cytotoxic representatives, whereas Tregs are a proxy for regulatory immunity [36]. Studies have shown the CD8T to Treg ratio as a significant biomarker for chemo- and immunotherapy responses [36, 38]. Our analyses with *HiTIMED* on TCGA showed better 5-year survival rates with higher CD8T memory cell levels in lung adenocarcinoma and better long-term survival in liver hepatocellular carcinoma, head and neck squamous cell carcinoma, and endocervical adenocarcinoma, which are consistent with its cytotoxic role in anti-tumoral activities. In kidney clear cell renal cell carcinoma, a higher level of Treg is associated with a worse survival outcome, indicating its role in immunosuppression [36]. Interestingly, in endometrial carcinoma, we observed significantly better survival with a higher level of Treg. This finding is consistent with a previous report on Treg being beneficial for survival in endometrial carcinoma [44]. The impact of Treg in cancer survival varies greatly by tumor site, suggesting differential physiological functions and roles of Tregs in different tumor types [45]. Based on TME composition, immune hot tumors are defined as tumors with a high level of immune cell infiltration and, thus, more likely to respond to immunotherapy [6, 42]. In our analysis, the unsupervised dichotomous classification of TCGA tumors by *HiTIMED* immune projection demonstrated the potential identification of immune hot and cold tumors. Future supervised training on paired data on immunotherapy response with *HiTIMED* immune projection promises a potential on systematically rating a tumor for immunotherapy response rate.

The angiogenic microenvironment supports tumor proliferation and metastasis [46]. The formation of new blood vessels relies heavily on endothelial and stromal cell proliferation [7]. In our study, a higher level of endothelial and stromal cells identified by *HiTIMED* was associated with worse survival rates in multiple cancers. Interestingly, in kidney clear cell renal cell carcinoma, a higher level of endothelial cells is beneficial for survival. This result is consistent with a single-cell analysis on kidney clear cell carcinoma, showing a better survival outcome in tumors with more endothelium [47]. A unique role of endothelial cells in prognostication of survival and immunotherapy response in kidney clear cell renal cell carcinoma patients has been hypothesized [47]. Worse 5-year survival outcomes were observed in multiple cancers for angiogenic hot tumors compared to angiogenic cold tumors in our analyses. Interestingly, immune hot and cold tumors were not significantly associated with 5-year survival after adjusting for age, gender, and tumor stage. Taken together, these data lead us to hypothesize that there is a closer relationship between the angiogenic microenvironment in TME with prognosis.

The cell type heterogeneity in TME complicates epidemiological analyses of TME and clinical outcomes. The association between cell type prevalence in TME and patient survival has previously been studied primarily by counting certain cells in TME using immunohistochemical quantification [29]. However, the cells in TME are dynamically interactive, making such analysis susceptible to other cell type confounders. The high resolution of *HiTIMED* makes it possible to adjust for such cell type confounders. Further, traditional EWAS analyses are susceptible to the cell type heterogeneity confounding. For instance, EWAS can identify valuable epigenetic biomarkers for early cancer detection and prognosis [52]. However, the sensitivity and precision of identifying such biomarkers are compromised when the tissue cell heterogeneity is ignored [53]. *HiTIMED*-projected cell composition in TME provides new opportunities for EWAS studies to unveil cell-type independent epigenetic biomarkers in cancer. Our results clearly show that much of the vast DNA methylation dysregulation previously observed in tumors is attributable to cell heterogeneity. Further application of *HiTIMED* cell estimates to models that identify tumor-specific DNA methylation is poised to enable a clearer understanding of early DNA methylation drivers alterations in carcinogenesis and disease progression.

While *HiTIMED* points to a valid method for estimating cell proportions in TME and the potential application to cancer research, we recognize some limitations. First, *HiTIMED* shows modest over-prediction for CD8T cells and under-prediction for CD4T cells, especially Tregs, in artificial mixtures. The *HiTIMED* libraries were developed to optimize the deconvolution in specific tumor microenvironments. We posited that the bias we observed in artificial mixtures would be minimized in specifically targeted tumor types. However, the hypothesis is hard to examine without known cell composition in TME. When collapsing the subtypes of T cells, *HiTIMED* is highly accurate, even in artificial mixtures. Also, immune cells are possibly reprogrammed by interaction with the TME. Thus using normal cells as a reference may generate noise. Second, the stomach adenocarcinoma showed least methylation distinction between tumor versus normal tissues compared to other carcinomas. This may attribute to the heterogeneity of stomach tumor cell subtypes. Future work on stomach cancer subtype specific libraries may be necessary for stomach TME deconvolution. Third, macrophages were not included in *HiTIMED.* Macrophage is a highly heterogeneous cell type in TME [48, 49]. Our initial effort on including macrophage generates substantial noise that confounds other mononuclear cells. Future effort on epigenetically defining tumor specific macrophages may help to address the issue. Fourth, only carcinomas are currently included in *HiTIMED,* and future work is needed to add other complex tumor types. Fifth, tumor subclones were not captured in all existing deconvolution methods. Future epigenetic data on tumor subclones guarantee a more extensive deconvolution of TME. Finally, the angiogenic/non-immune microenvironment profiled by *HiTIMED* cannot distinguish normal and tumor-impacted epithelial, endothelial, and stromal cells. However, our *HiTIMED-*profiled angiogenic microenvironment reflects angiogenesis globally, providing relevant information. Differentiating TME affected cells and normal cells may provide additional research avenues beyond the scope of this method.

## Conclusions

In summary, we developed *HiTIMED*, a DNA-methylation-based method to deconvolve the TME. This approach employs a novel tumor-type-specific hierarchical model with optimized libraries for each layer of deconvolution in each tumor type. *HiTIMED* provides higher cell type resolution compared to other methods, providing new opportunities to study the relation of the TME with etiologic factors, disease progression, and response to therapy.

## Methods

### Discovery data sets

For the discovery of our tumor TME deconvolution libraries, we used nine publicly available data sets from TCGA, Gene Expression Omnibus (GEO), and ArrayExpress, and two data sets from our laboratories available through GEO (GSE193297, GSE167998) that contain DNA methylation microarray data on 20 types of carcinomas and their matched normal, 12 types of purified immune cell, and three types of angiogenic cell (Additional file 1: Table S1) [50–53]. Purified basophils, eosinophils, neutrophils, monocytes, B naïve cells, B memory cells, CD4 naïve cells, CD4 memory cells, T regulatory cells, CD8 naïve cells, CD8 memory cells were cytometric and magnetic-sorted and flow confirmed. The artificial mixtures were generated from MACS-isolated and FACS-verified cells. The cells were purchased from AllCells® corporation (Alameda, CA, USA), StemExpress (Folsom, CA), and STEMCELL Technologies (Vancouver, BC, Canada). The donors included 41 males and 15 females, with a mean age of 32.2 years (sd = 12.2) and multiple ethnicities including African-Americans, East-Asian, Indo-European, and multiple/admixed. The donors were anonymous and healthy. For more details on sample information and preparation, please refer to our previous publication [12]. Dendritic cells used in this study were monocyte-derived dendritic cells from healthy human blood donors. Firstly, the PBMCs were isolated from buffy coat cells by Fiscoll density gradient centrifugation. Next, the CD14 cells were purified using immunomagnetic purification. Finally, 5-day incubation with 500 U/ml human granulocyte-macrophage colony-stimulating factor (hGM-CSF) (PeproTech, Rocky Hill, NJ) and 1,000 U/ml human interleukin 4 (hIL-4) (PeproTech, Rocky Hill, NJ) completed the procedure. More details on the protocol and procedure can be found at [54] and [55]. Although the discovery data sets contain Illumina HumanMethylation450k or HumanMethylationEPIC array data, to ensure the applicability of the library, we retained CpGs that were common to both platforms. Furthermore, cross-reactive probes, SNP-related probes, sex chromosome probes, and non-CpG probes were masked in the analysis. 384,640 CpGs were retained after this process. The *SeSAMe* pipeline from Bioconductor was used to preprocess the data, including data normalization and quality control [56]. The probes that contained over 20% of low-quality data (pOOBHA > 0.05) across samples per tissue type were removed for quality control.

### *HiTIMED* development

Due to the complexity and cell heterogeneity of TME, we propose a novel, tumor-type-specific hierarchical model to develop libraries with optimized accuracy for cell projection. In each tumor type, six layers of libraries were developed to hierarchically project cell proportions in first, tumor; second, angiogenic; and third, immune microenvironments (Fig. 1). For tumor purity estimation, the *InfiniumPurify* pipeline was employed to estimate the tumor purity [19]. The method identifies the top 1000 informative differentially methylated CpG (iDMC) sites between tumor and normal samples by rank-sum test and require that their variances of beta values are greater than 0.005 in tumor samples. The number 1000 was selected based on the performance of iterations of various number of iDMCs (50, 100, 200,500, 1000, 3000, 5000, 10,000, 15,000, 20,000, 30,000, 40,000). The performance was evaluated by correlating iDMC estimated purity and ABSOLUTE purity [25], which is somatic copy-number-based tumor purity estimation, in lung adenocarcinoma [19]. iDMCs were separated into hyper- and hypo-methylated groups based on their mean beta values in tumor and normal samples. The beta values for hypermethylated iDMCs remain unchanged whereas the hypomethylated iDMC beta values were transformed to 1-beta. Density estimation with Gaussian kernel was applied to the transformed iDMC beta values. The estimated purity is the mode of the density function. More details on *InfiniumPurify* pipeline can be found at [19]. In our study, we updated the pipeline by identifying tumor type specific iDMCs. Briefly, instead of using a universal set of iDMCs for estimating tumor purity for all tumor types, for each carcinoma type included in the study, we developed iDMCs specifically for that tumor type for tumor purity estimation. Epithelial, endothelial, stromal, basophil, eosinophil, neutrophil, monocyte, dendritic, B naïve, B memory, CD4 naïve, CD4 memory, T regulatory, CD8 naïve, CD8 memory cell proportions were estimated using the constrained projection/quadratic programming approach developed by Houseman et al. [21]. Libraries for specific cell types were developed using *limma* linear regression with empirical Bayes adjustment statistics in *Meffil* [20] to reduce methylation profiles to top 100 cell-type-specific hyper- and hypo-methylated CpGs. The number 100 was selected based on the performance of iterations of various number of cell type specific CpGs (50, 100, 200, 500, 1000). The performance was evaluated by calculating cell type specific absolute error and overall absolute error in colon adenocarcinoma (Additional file 2: Figure S17). The overall absolute error was minimal when using the 50-CpG library, however it had the worst performance in CD4 memory cell and eosinophils. To balance the performance across all cell types, we decided to use the 100-CpG library. The overall absolute error for 100-CpG library was only 0.2% lower than the 50-CpG library, however unlike the top-50 CpG library, the 100-CpG library did not have the worst performance across any of the cell types. More details on the hierarchical library construction can be found in the Results section and Fig. 1.

### Validation of *HiTIMED* projections

*HiTIMED* predicted tumor cell proportions were compared to the estimated tumor purity from major existing methods, including methylation-based *InfiniumPurify* [19]*, MethylCIBERSORT* [17]*, MethylResolver* [16]*, LUMP* [23]*,* gene expression-based *ESTIMATE* [24]*,* somatic copy-number-based *ABSOLUTE* [25]*,* image stain-based *IHC* [26]*,* and a consensus measurement of purity estimations (CPE) [26], using TCGA tumor data. One additional data sets of high-grade serous ovarian cancer was added due to the limited ovarian cancer sample size on TCGA (Additional file 1: Table S2) [57]. Tumor type stratified comparison between *HiTIMED* tumor proportion and *InfiniumPurify* tumor purity was conducted with Pearson's correlation coefficient, and the p-value was reported. Method paired pan-cancer tumor projection comparison was performed across *HiTIMED, MethylCIBERSORT, MethylResolver, CPE, ESTIMATE, LUMP, IHC,* and *ABSOLUTE*, with r and p-value reported. We applied *HiTIMED* to 12 artificial mixture samples with 12 predefined immune cell proportions (Additional file 1: Table S2). RMSE, R, and p-value were calculated for each of the 12 immune cell types by contrasting the *HiTIMED* cell estimates versus each sample's known ground truth proportion. To validate the angiogenic/non-immune microenvironment projection, *HiTIMED* was applied to publicly available normal human intestinal epithelium [27] and human umbilical vein endothelial cells [28] (Additional file 1: Table S2). Mean and standard deviation of *HiTIMED* predicted endothelial proportion and epithelial proportion were reported for normal human intestinal epithelium and human umbilical vein endothelial cells respectively.

### *HiTIMED* deconvolution compared to *MethylCIBERSORT* and *MethylResolver*

A Venn diagram was used to compare the cell types in the tumor microenvironment that can be captured by *HiTIMED, MethylCIBERSORT* and *MethylResolver.* All three methods were employed on the 12 immune cell artificial mixture samples for performance comparison. For cell types that can be estimated by all three methods, a performance comparison with operated by cell type and with all cells pooled. The error rate was calculated as$Predicted Proportion\left(\%\right)-True Proportion (\%)$. The absolute error rate was calculated as $|Predicted Proportion\left(\%\right)-True Proportion \left(\%\right)|$.

### Statistical analysis of the variation of TMEs and survival in TCGA samples

In TCGA samples, variances of immune and angiogenic microenvironments were calculated per tumor type. Tumor types were ranked by the variance of the immune microenvironment and angiogenic microenvironment, respectively, to demonstrate the across-tumor-type variation of TMEs. Ovarian cancer was removed from this analysis due to the limited sample size with survival information. Major immune cells (Bmem, CD8mem, DC, Tregs) and angiogenic cells (epithelial, endothelial, stromal) were investigated for 5-year survival outcomes in higher than median value group compared to lower than or equal to median value group across tumors using Cox proportional hazard models with age, gender, tumor proportion, tumor stage, and other cell-type proportions (Treg, Bmem, DC, CD8mem, epithelial, endothelial, stromal) adjusted. Two Cox models, with and without cell-type adjustment, were compared in clear cell renal cell carcinoma as sensitivity analyses. Gender-specific and tumor stage information unavailable cancer types were excluded from the survival analysis. The Schoenfeld residuals were used to test the proportional hazard assumption for Cox models. To ensure that the proportional hazard assumption was not violated in the Cox models, tumor stage was stratified into high stage and low stage in lung adenocarcinoma. Age was stratified into ten groups in the bladder carcinoma data set.

### Classification of immune and angiogenic hot/cold tumors and survival in TCGA samples

With the high resolution of *HiTIMED* predicted cell types, immune and hot tumors were classified using the consensus PAM clustering method based on *HiTIMED* projected granulocyte, mononuclear, T cell, B cell, and NK cell proportions in TCGA samples. Similarly, consensus PAM clustering was used to classify angiogenic hot and cold tumors based on *HiTIMED* projected epithelial, endothelial, and stromal cell proportions. Multivariable linear regression adjusting for age, gender, and tumor type, was used to compare *HiTIMED* projected cell proportions between immune/angiogenic hot and cold tumors. Cox proportional hazard models with age, gender, and tumor stage-adjusted were applied to investigate the survival outcomes in immune hot vs. cold tumors and angiogenic hot vs. cold tumors. Cancer types gender-specific and with tumor stage information unavailable were excluded from this analysis. The proportional hazards assumption of all models was checked using the Schoenfeld residuals test. Log-rank tests were used to test survival differences in four groups of tumor clusters that were generated by combining the immune and angiogenic hot and cold classification. The Student's t-test was used to compare *HiTIMED* immune cells between immune subtyped C2 and C6 tumors [30].

### Models comparing methylation profile between colon adenocarcinoma and adjacent normal samples

Three models were generated to identify DMCs between colon adenocarcinoma and normal adjacent tissues. Model 1 adjusted for age and gender. Model 2 adjusted for age, gender, and *HiTIMED-*projected tumor purity. Model 3 adjusted for age, gender, *HiTIMED-*projected tumor purity, DC, CD8mem, Bmem, Treg, epithelial, endothelial, and stromal cell proportions. Delta betas larger than 0.3 and FDR smaller than 0.01 were used as the cut-off for statistically significant DMC identification. Heatmaps with Manhattan distance clustering and colon cancer CIMP subtypes colored were generated per model.

### Statistical analysis on TMEs in drug-sensitive and resistant mCRC and recurrent TNBC

TMEs in drug-sensitive and resistant mCRC and recurrent TNBC were deconvolved using *HiTIMED*. Student's t-tests were used to compare the means of 17 *HiTIMED* projected cell types between first-line chemotherapy drug-sensitive and resistant mCRC tumors. Similarly, Student's t-tests were also used to compare the means of 17 *HiTIMED* projected cell types between recurrent and non-recurrent TNBC tumors in the chemotherapy-treated arm and nonchemotherapy-treated arm after locoregional therapy, respectively.

## Supplementary Information

**Additional file 1: Table S1.** Baseline characteristics of the discovery data sets. **Table S2.** Baseline characteristics of the validation and application data sets. **Table S3.** Statistically significant hazard ratios of *HiTIMED*-projected cell proportions in cancer patients' 5-year survival adjusted for age, gender, tumor stage, *HiTIMED*-projected tumor proportion, and other cell-type proportions.

**Additional file 2: Figure S1.** Correlation between *HiTIMED *tumor and *InfiniumPurify *tumor by tumor type across cholangiocarcinoma, kidney papillary cell carcinoma, pancreatic adenocarcinoma, and stomach adenocarcinoma. **Figure S2.** Methylation state of CpGs in the *HiTIMED *tumor specific library (L1) and *InfiniumPurify *default library. **Figure S3.** *HiTIMED *tumor purity vs *InfiniumPurify *tumor purity in thyroid carcinoma. **Figure S4.** *HiTIMED *tumor proportion vs other method predicted tumor proportion. **Figure S5.** *HiTIMED *immune cell proportions vs true immune cell proportions in artificial mixtures. **Figure S6.** *HiTIMED *T cell proportion vs true T cell proportion in artificial mixtures. **Figure S7.** *HiTIMED* cell composition in human normal intestinal epithelium and umbilical vein endothelial cells. **Figure S8. **Performance comparison across *HiTIMED, MethylCIBERSORT, and MethylResolver *using artificial mixtures. **Figure S9.** The distribution of the *HiTIMED *cell composition in TCGA tumors. **Figure S10.** Cell composition differs substantially and captures sample heterogeneity using *HiTIMED*-projected proportions. Seventeen cell types were captured for each sample by tumor type. **Figure S11**. Sensitive analysis comparing outputs from two Cox models with or without cell type proportions adjusted in kidney clear cell carcinoma. **Figure S12.** Kaplan-Meier survival curves for *HiTIMED *cells estimates in TCGA tumors. **Figure 13.** *HiTIMED* cell comparison and Kaplan-Meier survival curves across immune/angiogenic hot and cold. **Figure S14.** *HiTIMED* immune and angiogenic proportions across C1-C6 subtyped TCGA tumor. **Figure S15.** *HiTIMED* cell comparisons between drug-sensitive and -resistant metastasized colorectal cancer. **Figure S16.** *HiTIMED *cell comparisons in triple−negative breast cancer w/without chemotherapy. **Figure S17.** Performance comparison across iterations on CpGs selected in *HiTIMED *for immune and angiogenic cell projection.

## Data Availability

All data sets used in this study are publicly available on The Cancer Genome Atlas (TCGA), Gene Expression Omnibus (GEO), and ArrayExpress. The accession numbers are GSE46306, GSE146552, GSE53051, GSE164988, GSE122126, GSE133556, GSE182379, GSE82234, GSE148766, GSE100503, GSE66313, GSE141441, GSE167998, GSE193297, E-MTAB-3027, E-MTAB-8952, E-MTAB-5463.

## Abbreviations

TME: Tumor microenvironment
DC: Dendritic cell
Mono: Monocyte
Bas: Basophil
Eos: Eosinophil
Neu: Neutrophil
NK: Natural killer cell
Bnv: B naïve cell
Bmem: B memory cell
CD4nv: CD4 naïve cell
CD4mem: CD4 memory cell
Treg: T regulatory cell
CD8nv: CD8 naïve cell
CD8mem: CD8 memory cell
BLCA: Bladder urothelial carcinoma
BRCA: Breast invasive carcinoma
CESC: Cervical squamous cell carcinoma and endocervical adenocarcinoma
CHOL: Cholangiocarcinoma
COAD: Colon adenocarcinoma
ESCA: Esophageal carcinoma
HNSC: Head and neck squamous cell carcinoma
KIRC: Kidney clear cell renal cell carcinoma
LIHC: Liver hepatocellular carcinoma
LUAD: Lung adenocarcinoma
LUSC: Lung squamous cell carcinoma
PAAD: Pancreatic adenocarcinoma
PRAD: Prostate adenocarcinoma
READ: Rectum adenocarcinoma
STAD: Stomach adenocarcinoma
THCA: Thyroid carcinoma
UCEC: Uterine corpus endometrial carcinoma
TCGA: The Cancer Genome Atlas
TNBC: Triple-negative breast cancer

## References

[1] Labani-Motlagh A, Ashja-Mahdavi M, Loskog A (2020). The tumor microenvironment: a milieu hindering and obstructing antitumor immune responses. Front Immunol.

[2] Jin MZ, Jin WL (2020). The updated landscape of tumor microenvironment and drug repurposing. Signal Transduct Target Ther.

[3] Baghban R, Roshangar L, Jahanban-Esfahlan R, Seidi K, Ebrahimi-Kalan A, Jaymand M (2020). Tumor microenvironment complexity and therapeutic implications at a glance. Cell Commun Signal.

[4] Thiery JP, Chopin D (1999). Epithelial cell plasticity in development and tumor progression. Cancer Metastasis Rev.

[5] Wang H, Li S, Wang Q, Jin Z, Shao W, Gao Y (2021). Tumor immunological phenotype signature-based high-throughput screening for the discovery of combination immunotherapy compounds. Sci Adv.

[6] Duan Q, Zhang H, Zheng J, Zhang L (2020). Turning cold into hot: firing up the tumor microenvironment. Trends Cancer.

[7] Sewduth R, Santoro MM (2016). "Decoding" angiogenesis: new facets controlling endothelial cell behavior. Front Physiol.

[8] El-Kenawi AE, El-Remessy AB (2013). Angiogenesis inhibitors in cancer therapy: mechanistic perspective on classification and treatment rationales. Br J Pharmacol.

[9] Newman AM, Liu CL, Green MR, Gentles AJ, Feng W, Xu Y (2015). Robust enumeration of cell subsets from tissue expression profiles. Nat Methods.

[10] Bogdanovic O, Lister R (2017). DNA methylation and the preservation of cell identity. Curr Opin Genet Dev.

[11] Titus AJ, Gallimore RM, Salas LA, Christensen BC (2017). Cell-type deconvolution from DNA methylation: a review of recent applications. Hum Mol Genet.

[12] Salas LA, Zhang Z, Koestler DC, Butler RA, Hansen HM, Molinaro AM (2022). Enhanced cell deconvolution of peripheral blood using DNA methylation for high-resolution immune profiling. Nat Commun.

[13] Salas LA, Koestler DC, Butler RA, Hansen HM, Wiencke JK, Kelsey KT (2018). An optimized library for reference-based deconvolution of whole-blood biospecimens assayed using the Illumina HumanMethylationEPIC beadarray. Genome Biol.

[14] Salas LA, Lundgren SN, Browne EP, Punska EC, Anderton DL, Karagas MR (2020). Prediagnostic breast milk DNA methylation alterations in women who develop breast cancer. Hum Mol Genet.

[15] Muse ME, Bergman DT, Salas LA, Tom LN, Tan JM, Laino A (2021). Genome-scale DNA methylation analysis identifies repeat element alterations that modulate the genomic stability of melanocytic nevi. J Invest Dermatol.

[16] Arneson D, Yang X, Wang K (2020). MethylResolver-a method for deconvoluting bulk DNA methylation profiles into known and unknown cell contents. Commun Biol.

[17] Chakravarthy A, Furness A, Joshi K, Ghorani E, Ford K, Ward MJ (2018). Pan-cancer deconvolution of tumour composition using DNA methylation. Nat Commun.

[18] Paz MF, Fraga MF, Avila S, Guo M, Pollan M, Herman JG (2003). A systematic profile of DNA methylation in human cancer cell lines. Cancer Res.

[19] Zheng X, Zhang N, Wu HJ, Wu H (2017). Estimating and accounting for tumor purity in the analysis of DNA methylation data from cancer studies. Genome Biol.

[20] Min JL, Hemani G, Davey Smith G, Relton C, Suderman M (2018). Meffil: efficient normalization and analysis of very large DNA methylation datasets. Bioinformatics.

[21] Houseman EA, Accomando WP, Koestler DC, Christensen BC, Marsit CJ, Nelson HH (2012). DNA methylation arrays as surrogate measures of cell mixture distribution. BMC Bioinformatics.

[22] Amendoeira I, Maia T, Sobrinho-Simoes M (2018). Non-invasive follicular thyroid neoplasm with papillary-like nuclear features (NIFTP): impact on the reclassification of thyroid nodules. Endocr Relat Cancer.

[23] Benelli M, Romagnoli D, Demichelis F (2018). Tumor purity quantification by clonal DNA methylation signatures. Bioinformatics.

[24] Yoshihara K, Shahmoradgoli M, Martinez E, Vegesna R, Kim H, Torres-Garcia W (2013). Inferring tumour purity and stromal and immune cell admixture from expression data. Nat Commun.

[25] Carter SL, Cibulskis K, Helman E, McKenna A, Shen H, Zack T (2012). Absolute quantification of somatic DNA alterations in human cancer. Nat Biotechnol.

[26] Aran D, Sirota M, Butte AJ (2015). Systematic pan-cancer analysis of tumour purity. Nat Commun.

[27] Howell KJ, Kraiczy J, Nayak KM, Gasparetto M, Ross A, Lee C (2018). DNA methylation and transcription patterns in intestinal epithelial cells from pediatric patients with inflammatory bowel diseases differentiate disease subtypes and associate with outcome. Gastroenterology.

[28] Franzen J, Zirkel A, Blake J, Rath B, Benes V, Papantonis A (2017). Senescence-associated DNA methylation is stochastically acquired in subpopulations of mesenchymal stem cells. Aging Cell.

[29] Craig SG, Humphries MP, Alderdice M, Bingham V, Richman SD, Loughrey MB (2020). Immune status is prognostic for poor survival in colorectal cancer patients and is associated with tumour hypoxia. Br J Cancer.

[30] Thorsson V, Gibbs DL, Brown SD, Wolf D, Bortone DS, Ou Yang TH (2018). The immune landscape of cancer. Immunity.

[31] Xu Z, Bolick SC, DeRoo LA, Weinberg CR, Sandler DP, Taylor JA (2013). Epigenome-wide association study of breast cancer using prospectively collected sister study samples. J Natl Cancer Inst.

[32] Verma M (2012). Epigenome-wide association studies (EWAS) in cancer. Curr Genomics.

[33] Shenker NS, Polidoro S, van Veldhoven K, Sacerdote C, Ricceri F, Birrell MA (2013). Epigenome-wide association study in the European prospective investigation into cancer and nutrition (EPIC-Turin) identifies novel genetic loci associated with smoking. Hum Mol Genet.

[34] Wang Z, Lu Y, Fornage M, Jiao L, Shen J, Li D (2022). Identification of novel susceptibility methylation loci for pancreatic cancer in a two-phase epigenome-wide association study. Epigenetics.

[35] Chen B, Khodadoust MS, Liu CL, Newman AM, Alizadeh AA (2018). Profiling tumor infiltrating immune cells with CIBERSORT. Methods Mol Biol.

[36] Kareva I (2019). Metabolism and gut microbiota in cancer immunoediting, CD8/Treg ratios, immune cell homeostasis, and cancer (immuno)therapy: concise review. Stem Cells.

[37] Klebanoff CA, Gattinoni L, Restifo NP (2006). CD8+ T-cell memory in tumor immunology and immunotherapy. Immunol Rev.

[38] Baras AS, Drake C, Liu JJ, Gandhi N, Kates M, Hoque MO (2016). The ratio of CD8 to Treg tumor-infiltrating lymphocytes is associated with response to cisplatin-based neoadjuvant chemotherapy in patients with muscle invasive urothelial carcinoma of the bladder. Oncoimmunology.

[39] Pham TN, Hong CY, Min JJ, Rhee JH, Nguyen TA, Park BC (2010). Enhancement of antitumor effect using dendritic cells activated with natural killer cells in the presence of toll-like receptor agonist. Exp Mol Med.

[40] Liu B, Yang X, Wang T, Lin J, Kang Y, Jia P (2019). MEpurity: estimating tumor purity using DNA methylation data. Bioinformatics.

[41] Hennessey PT, Ochs MF, Mydlarz WW, Hsueh W, Cope L, Yu W (2011). Promoter methylation in head and neck squamous cell carcinoma cell lines is significantly different than methylation in primary tumors and xenografts. PLoS ONE.

[42] Liu YT, Sun ZJ (2021). Turning cold tumors into hot tumors by improving T-cell infiltration. Theranostics.

[43] Bonaventura P, Shekarian T, Alcazer V, Valladeau-Guilemond J, Valsesia-Wittmann S, Amigorena S (2019). Cold tumors: a therapeutic challenge for immunotherapy. Front Immunol.

[44] Liu W, Sun L, Zhang J, Song W, Li M, Wang H (2021). Biosci Rep.

[45] Shang B, Liu Y, Jiang SJ, Liu Y (2015). Prognostic value of tumor-infiltrating FoxP3+ regulatory T cells in cancers: a systematic review and meta-analysis. Sci Rep.

[46] Zetter BR (1998). Angiogenesis and tumor metastasis. Annu Rev Med.

[47] Zhang Y, Narayanan SP, Mannan R, Raskind G, Wang X, Vats P (2021). Single-cell analyses of renal cell cancers reveal insights into tumor microenvironment, cell of origin, and therapy response. Proc Natl Acad Sci U S A.

[48] Komohara Y, Jinushi M, Takeya M (2014). Clinical significance of macrophage heterogeneity in human malignant tumors. Cancer Sci.

[49] Wu MF, Lin CA, Yuan TH, Yeh HY, Su SF, Guo CL (2021). The M1/M2 spectrum and plasticity of malignant pleural effusion-macrophage in advanced lung cancer. Cancer Immunol Immunother.

[50] Farkas SA, Milutin-Gasperov N, Grce M, Nilsson TK (2013). Genome-wide DNA methylation assay reveals novel candidate biomarker genes in cervical cancer. Epigenetics.

[51] Zhang W, Klinkebiel D, Barger CJ, Pandey S, Guda C, Miller A (2020). Global DNA hypomethylation in epithelial ovarian cancer: passive demethylation and association with genomic instability. Cancers (Basel).

[52] Timp W, Bravo HC, McDonald OG, Goggins M, Umbricht C, Zeiger M (2014). Large hypomethylated blocks as a universal defining epigenetic alteration in human solid tumors. Genome Med.

[53] Moss J, Magenheim J, Neiman D, Zemmour H, Loyfer N, Korach A (2018). Comprehensive human cell-type methylation atlas reveals origins of circulating cell-free DNA in health and disease. Nat Commun.

[54] Nair S, Archer GE, Tedder TF (2012). Isolation and generation of human dendritic cells. Curr Protoc Immunol.

[55] Hartmann BM, Thakar J, Albrecht RA, Avey S, Zaslavsky E, Marjanovic N (2015). Human dendritic cell response signatures distinguish 1918, pandemic, and seasonal H1N1 influenza viruses. J Virol.

[56] Zhou W, Triche TJ, Laird PW, Shen H (2018). SeSAMe: reducing artifactual detection of DNA methylation by infinium beadchips in genomic deletions. Nucleic Acids Res.

[57] Gonzalez Bosquet J, Devor EJ, Newtson AM, Smith BJ, Bender DP, Goodheart MJ (2021). Creation and validation of models to predict response to primary treatment in serous ovarian cancer. Sci Rep.

