# Supplementary material for: HiTIMED: hierarchical tumor immune microenvironment epigenetic deconvolution for accurate cell type resolution in the tumor microenvironment using tumor-type-specific DNA methylation data
Source: J Transl Med. 2022 Nov 8;20:516. doi: 10.1186/s12967-022-03736-6 (PMC9644569; doi:10.1186/s12967-022-03736-6)
Supplement: Supplementary file 1 — Additional file 1: Table S1. Baseline characteristics of the discovery data sets. Table S2. Baseline characteristics of the validation and application data sets. Table S3. Statistically significant hazard ratios of HiTIMED-projected cell proportions in cancer patients' 5-year survival adjusted for age, gender, tumor stage, HiTIMED-projected tumor proportion, and other cell-type proportions. [file 12967_2022_3736_MOESM1_ESM.pdf]

Sample	N	Mean Age (SD)	Male N (%)	White N (%)	Black N (%)	Asian N (%)	Unreported Race N (%)	Other Race N (%)	Stage I N (%)	Stage II N (%)	Stage III N (%)	Stage IV N (%)	Unreported Stage N (%)	Data Source
Bladder urothelial carcinoma (muscle invasive)	412	68 (10.6)	304 (73.8)	327 (79.4)	23 (5.6)	44 (10.7)	18 (4.4)	0 (0)	2 (0.5)	131 (32.0)	141 (34.4)	136 (33.2)	2 (0.5)	TCGA
Normal bladder	21	69 (11.7)	11 (52.4)	18 (85.7)	2 (9.5)	0 (0)	1 (4.8)	0 (0)	--	--	--	--	--	TCGA
Breast invasive carcinoma	784	58 (13.2)	9 (1.1)	569 (72.6)	160 (20.4)	38 (4.8)	16 (2.0)	1 (0.1)	127 (16.5)	436 (56.5)	198 (25.6)	11 (1.4)	12 (1.5)	TCGA
Normal breast	97	58 (15.2)	0 (0)	91 (93.8)	4 (4.1)	1 (1.0)	1 (1.0)	0 (0)	--	--	--	--	--	TCGA
Cervical squamous cell carcinoma and endocervical adenocarcinoma	307	48 (13.79)	0 (0)	211 (68.7)	30 (9.8)	20 (6.5)	36 (11.7)	10 (3.3)	NA	NA	NA	NA	NA	TCGA
Normal cervix	20	35 (13.33)*	0 (0)	NA	NA	NA	NA	NA	--	--	--	--	--	GSE46306
Cholangiocarcinoma	36	63 (12.9)	16 (44.4)	31 (86.1)	2 (5.6)	3 (8.3)	0 (0)	0 (0)	19 (52.8)	9 (25.0)	1 (2.8)	7 (19.4)	0 (0)	TCGA
Normal bile duct	9	71 (8.9)	6 (66.7)	9 (100.0)	0 (0)	0 (0)	0 (0)	0 (0)	--	--	--	--	--	TCGA
Colon adenocarcinoma	284	65 (13.4)	149 (52.5)	194 (68.3)	58 (20.4)	11 (3.9)	20 (7.0)	1 (0.4)	43 (15.7)	109 (39.8)	81 (29.6)	41 (15.0)	10 (3.5)	TCGA
Normal colon	38	69 (12.4)	21 (55.3)	20 (52.6)	4 (10.5)	0 (0)	14 (36.8)	0 (0)	--	--	--	--	--	TCGA
Esophageal carcinoma	185	62 (11.9)	158 (85.4)	114 (61.6)	5 (2.7)	46 (24.9)	20 (10.8)	0 (0)	18 (11.1)	79 (48.8)	56 (34.6)	9 (5.6)	23 (12.4)	TCGA
Normal esophagus	16	68 (11.0)	10 (62.5)	16 (100.0)	0 (0)	0 (0)	0 (0)	0 (0)	--	--	--	--	--	TCGA
Head and neck squamous cell carcinoma	528	61 (11.9)	386 (73.1)	452 (85.6)	48 (9.1)	11 (2.1)	15 (2.8)	2 (0.4)	27 (6.0)	74 (16.3)	82 (18.1)	270 (59.6)	75 (14.2)	TCGA
Normal head and neck tissue	50	62 (10.6)	38 (76.0)	43 (86.0)	6 (12.0)	0 (0)	1 (2.0)	0 (0)	--	--	--	--	--	TCGA
Kidney chromophobe carcinoma	66	52 (14.3)	39 (59.1)	58 (87.9)	4 (6.1)	2 (3.0)	2 (3.0)	0 (0)	21 (31.8)	25 (37.9)	14 (21.2)	6 (9.1)	0 (0)	TCGA
Kidney renal clear cell carcinoma	319	61 (11.8)	205 (64.3)	266 (83.4)	49 (15.4)	1 (0.3)	3 (0.9)	0 (0)	155 (48.9)	31 (9.8)	73 (23.0)	58 (18.3)	2 (0.6)	TCGA
Kidney renal papillary cell carcinoma	275	62 (12.1)	202 (73.5)	198 (72.0)	54 (19.6)	6 (2.2)	15 (5.5)	2 (0.7)	168 (66.9)	18 (7.2)	51 (20.3)	14 (5.6)	24 (8.7)	TCGA
Normal kidney	205	63 (11.7)	137 (66.8)	182 (88.8)	14 (6.8)	1 (0.5)	7 (3.4)	1 (0.5)	--	--	--	--	--	TCGA
Liver hepatocellular carcinoma	377	59 (13.5)	255 (67.6)	187 (49.6)	17 (4.5)	161 (42.7)	10 (2.7)	2 (0.5)	175 (49.6)	87 (24.6)	86 (24.4)	5 (1.4)	24 (6.4)	TCGA
Normal liver	50	62 (16.0)	30 (60.0)	34 (68.0)	7 (14.0)	6 (12.0)	3 (6.0)	0 (0)	--	--	--	--	--	TCGA
Lung adenocarcinoma	458	65 (10.2)	214 (46.7)	353 (77.1)	50 (10.9)	6 (1.3)	49 (10.7)	0 (0)	248 (54.7)	112 (24.7)	73 (16.1)	20 (4.4)	5 (1.1)	TCGA
Lung squamous cell carcinoma	370	68 (8.7)	274 (74.1)	274 (74.1)	24 (6.5)	7 (1.9)	65 (17.6)	0 (0)	172 (46.9)	135 (36.8)	56 (15.3)	4 (1.1)	3 (0.8)	TCGA
Normal lung	74	67 (10.8)	46 (62.2)	60 (81.1)	6 (8.1)	1 (1.4)	7 (9.5)	0 (0)	--	--	--	--	--	TCGA
Ovarian serous cystadenocarcinoma	10	62 (9.1)	0 (0)	0 (0)	10 (100.0)	0 (0)	0 (0)	0 (0)	NA	NA	NA	NA	NA	TCGA
Normal ovarian tissue	18	43 (10.6)*	0 (0)	13 (72.2)#	5 (27.8)#	0 (0)#	0 (0)#	0 (0)#	--	--	--	--	--	GSE146552
Pancreatic adenocarcinoma	29	60 (15.4)*	NA	NA	NA	NA	NA	NA	NA	NA	NA	NA	NA	GSE53051
Normal pancreas	12	54 (12.2)*	NA	NA	NA	NA	NA	NA	--	--	--	--	--	GSE53051
Prostate adenocarcinoma	498	61 (6.8)	498 (100.0)	413 (82.9)	58 (11.6)	12 (2.4)	14 (2.8)	1 (0.2)	NA	NA	NA	NA	NA	TCGA
Normal prostate	50	62 (6.5)	50 (100.0)	47 (94.0)	0 (0)	0 (0)	3 (6.0)	0 (0)	--	--	--	--	--	TCGA
Rectum adenocarcinoma	98	63 (12.3)	53 (54.1)	76 (77.6)	5 (5.1)	1 (1.0)	16 (16.3)	0 (0)	11 (12.5)	29 (33.0)	35 (39.8)	13 (14.8)	10 (10.2)	TCGA
Normal rectum	12	68 (9.9)	5 (41.7)	1 (8.3)	0 (0)	0 (0)	7 (58.3)	4 (33.3)	--	--	--	--	--	TCGA, E-MTAB-3027
Stomach adenocarcinoma	12	59 (20.9)*	7 (58.3)#	0 (0)#	0 (0)#	12 (100)#	0 (0)#	0 (0)#	NA	NA	NA	NA	NA	GSE164988
Normal stomach	12	65 (6.6)*	7 (58.3)#	0 (0)#	0 (0)#	12 (100)#	0 (0)#	0 (0)#	--	--	--	--	--	GSE164988
Thyroid carcinoma	507	47 (15.8)	136 (26.8)	334 (65.9)	27 (5.3)	52 (10.3)	93 (18.3)	1 (0.2)	285 (56.4)	52 (10.3)	113 (22.4)	55 (10.9)	2 (0.4)	TCGA
Normal Thyroid	56	46 (17.1)	14 (25.0)	38 (67.9)	6 (10.7)	3 (5.4)	9 (16.1)	9 (16.1)	--	--	--	--	--	TCGA
Uterine corpus endometrial carcinoma	431	64 (11.2)	0 (0)	287 (66.6)	98 (22.7)	8 (1.9)	9 (2.1)	29 (6.7)	NA	NA	NA	NA	NA	TCGA
Normal endometrial tissue	46	61 (11.3)	0 (0)	25 (54.3)	6 (13.0)	1 (2.2)	13 (28.3)	0 (0)	--	--	--	--	--	TCGA
Normal lung and colon epithelial cell	6	67 (12.4)	5 (83.3)	6 (100)#	0 (0)#	0 (0)#	0 (0)#	0 (0)#	--	--	--	--	--	GSE122126
Normal vascular and lymphatic endothelial cell	6	28 (29.0)*	4 (66.7)#	4 (66.7)#	0 (0)#	2 (33.3)#	0 (0)#	0 (0)#	--	--	--	--	--	GSE122126, E-MTAB-8952
Normal cord tissue stromal cell	14	0 (0)	10 (71.4)	5 (35.7)	4 (28.6)	5 (35.7)	0 (0)	0 (0)	--	--	--	--	--	FlowSorted.CordTissueAndBlood.EPIC
Basophil	6	46 (14.0)	5 (83.3)	5 (83.3)	1 (16.7)	0 (0)	0 (0)	0 (0)	--	--	--	--	--	GSE167998
Eosinophil	4	25 (4.5)	3 (75.0)	0 (0)	1 (25.0)	3 (75.0)	0 (0)	0 (0)	--	--	--	--	--	GSE167998
Neutrophil	6	27 (7.3)	5 (83.3)	3 (50.0)	1 (16.7)	0 (0)	0 (0)	2 (33.3)	--	--	--	--	--	GSE167998
Monocyte	5	33 (9.8)	4 (80.0)	2 (40.0)	1 (20.0)	1 (20.0)	0 (0)	1 (20.0)	--	--	--	--	--	GSE167998
NK	4	34 (13.5)	4 (100.0)	3 (75.0)	1 (25.0)	0 (0)	0 (0)	0 (0)	--	--	--	--	--	GSE167998
B naïve	4	35 (5.3)	2 (50.0)	2 (50.0)	1 (25.0)	0 (0)	0 (0)	1 (25.0)	--	--	--	--	--	GSE167998
B memory	6	32 (12.0)	3 (50.0)	5 (83.3)	1 (16.7)	0 (0)	0 (0)	0 (0)	--	--	--	--	--	GSE167998
CD4T naïve	5	24 (2.3)	5 (100.0)	5 (100.0)	0 (0)	0 (0)	0 (0)	0 (0)	--	--	--	--	--	GSE167998
CD4T memory	4	41 (14.5)	3 (75.0)	4 (100.0)	0 (0)	0 (0)	0 (0)	0 (0)	--	--	--	--	--	GSE167998
T regulatory	3	34 (15.5)	1 (33.3)	2 (66.7)	1 (33.3)	0 (0)	0 (0)	0 (0)	--	--	--	--	--	GSE167998
CD8T naïve	5	29 (9.9)	4 (80.0)	5 (100.0)	0 (0)	0 (0)	0 (0)	0 (0)	--	--	--	--	--	GSE167998
CD8T memory	4	24 (2.3)	5 (100.0)	5 (100.0)	0 (0)	0 (0)	0 (0)	0 (0)	--	--	--	--	--	GSE167998
Dendritic cell	5	55 (12.8)*	2 (40)#	4 (80.0)#	0 (0)#	1 (20.0)#	0 (0)#	0 (0)#	--	--	--	--	--	GSE193297
Total	6859													

*Horvath methylation age inferred using *wateRmelon*

Inferred sex and ethnicity, based on methylation using *SeSAMe*

Supplementary Table 1

Data Source	Tissue Type	N	Mean Age (SD)	Male N (%)	White N (%)	Black N (%)	Asian N (%)	Unreported Race N (%)	Other Race N (%)
GSE133556	High grade serous ovarian cancer	99	43 (16.5)*	0 (0)	97 (98.0)#	1 (1.0)#	1 (1.0)#	0 (0)#	0 (0)#
GSE182379	Immune cell artificial mixtures	12	--	--	--	--	--	--	--
TCGA	Pancreatic adenocarcinoma	184	65 (11.0)	102 (55.4)	162 (88.0)	7 (3.8)	11 (6.0)	4 (2.2)	0 (0)
TCGA	Stomach adenocarcinoma	395	65 (10.7)	259 (65.6)	253 (64.1)	13 (3.3)	89 (22.5)	39 (9.9)	1 (0.3)
E-MTAB-5463	Normal human intestinal epithelium	6	10 (5.7)	1 (10.0)	6 (100)#	0 (0)#	0 (0)#	0 (0)#	0 (0)#
GSE82234	Human umbilical vein endothelial cells	6	0 (0)	6 (100)	4 (66.7)#	2 (33.3)#	0 (0)#	0 (0)#	0 (0)#
GSE148766	Drug sensitive and resistant metastatic colorectal carcinoma	36	50 (13.5)*	NA	NA	NA	NA	NA	NA
GSE100503	Breast in ductal carcinoma in situ core biopsy	13	56 (11.3)	13 (100)	13 (100)#	0 (0)#	0 (0)#	0 (0)#	0 (0)#
GSE66313	Breast in ductal carcinoma in situ surgical excision	13	56 (11.3)	13 (100)	13 (100)#	0 (0)#	0 (0)#	0 (0)#	0 (0)#
GSE141441	Triple-nengative breast cancer recurrence	166	59 (15.4)	0 (0)	NA	NA	NA	NA	NA

*Horvath methylation age inferred using *wateRmelon*

Inferred sex and ethnicity, based on methylation using *SeSAMe*

Tumor Type	Cell Type	Hazard ratio (95%CI)
Head and neck squamous cell carcinoma	Stromal	1.51 (1.02, 2.22)
	Endothelial	1.57 (1.07, 2.29)
Bladder carcinoma	Dendritic cell	0.45 (0.28, 0.73)
Kidney papillary carcinoma	Endothelial	3.48 (1.27, 9.55)
	Stromal	3.44 (1.46, 8.13)
Lung adenocarcinoma	CD8mem	0.50 (0.32, 0.79)
	Endothelial	1.83 (1.13, 2.95)
	Epithelial	0.50 (0.31, 0.80)
Lung squamous cell carcinoma	Epithelial	1.80 (1.16, 2.78)
Thyroid carcinoma	Bmem	7.13 (1.17, 43.53)

Supplementary Table 3
