# Supplementary material for: HiTIMED: hierarchical tumor immune microenvironment epigenetic deconvolution for accurate cell type resolution in the tumor microenvironment using tumor-type-specific DNA methylation data
Source: J Transl Med. 2022 Nov 8;20:516. doi: 10.1186/s12967-022-03736-6 (PMC9644569; doi:10.1186/s12967-022-03736-6)
Supplement: Supplementary file 2 — Additional file 2: Figure S1. Correlation between HiTIMED tumor and InfiniumPurify tumor by tumor type across cholangiocarcinoma, kidney papillary cell carcinoma, pancreatic adenocarcinoma, and stomach adenocarcinoma. Figure S2. Methylation state of CpGs in the HiTIMED tumor specific library (L1) and InfiniumPurify default library. Figure S3. HiTIMED tumor purity vs InfiniumPurify tumor purity in thyroid carcinoma. Figure S4. HiTIMED tumor proportion vs other method predicted tumor proportion. Figure S5. HiTIMED immune cell proportions vs true immune cell proportions in artificial mixtures. Figure S6. HiTIMED T cell proportion vs true T cell proportion in artificial mixtures. Figure S7. HiTIMED cell composition in human normal intestinal epithelium and umbilical vein endothelial cells. Figure S8. Performance comparison across HiTIMED, MethylCIBERSORT, and MethylResolver using artificial mixtures. Figure S9. The distribution of the HiTIMED cell composition in TCGA tumors. Figure S10. Cell composition differs substantially and captures sample heterogeneity using HiTIMED-projected proportions. Seventeen cell types were captured for each sample by tumor type. Figure S11. Sensitive analysis comparing outputs from two Cox models with or without cell type proportions adjusted in kidney clear cell carcinoma. Figure S12. Kaplan-Meier survival curves for HiTIMED cells estimates in TCGA tumors. Figure 13. HiTIMED cell comparison and Kaplan-Meier survival curves across immune/angiogenic hot and cold. Figure S14. HiTIMED immune and angiogenic proportions across C1-C6 subtyped TCGA tumor. Figure S15. HiTIMED cell comparisons between drug-sensitive and -resistant metastasized colorectal cancer. Figure S16. HiTIMED cell comparisons in triple−negative breast cancer w/without chemotherapy. Figure S17. Performance comparison across iterations on CpGs selected in HiTIMED for immune and angiogenic cell projection. [file 12967_2022_3736_MOESM2_ESM.pdf]

Supplementary Figures

HiTIMED: Hierarchical **T**umor **I**mmune **M**icroenvironment **E**pigenetic **D**econvolution for accurate cell type resolution in the tumor microenvironment using tumor-type-specific DNA methylation data

Table of Contents

Supplementary Figure 1. Correlation between *HiTIMED* tumor and *InfiniumPurify* tumor by tumor type across cholangiocarcinoma, kidney papillary cell carcinoma, pancreatic adenocarcinoma, and stomach adenocarcinoma 3

Supplementary Figure 2. Methylation state of CpGs in the *HiTIMED* tumor specific library (L1) and *InfiniumPurify* default library 4

Supplementary Figure 3. *HiTIMED* tumor purity vs *InfiniumPurify* tumor purity in thyroid carcinoma 5

Supplementary Figure 4. *HiTIMED* tumor proportion vs other method predicted tumor proportion 6

Supplementary Figure 5. *HiTIMED* immune cell proportions vs true immune cell proportions in artificial mixtures 7

Supplementary Figure 6. *HiTIMED* T cell proportion vs true T cell proportion in artificial mixtures 8

Supplementary Figure 7. *HiTIMED* cell composition in human normal intestinal epithelium and umbilical vein endothelial cells 9

Supplementary Figure 8. Performance comparison across *HiTIMED, MethylCIBERSORT, and MethylResolver* using artificial mixtures 10

Supplementary Figure 9. The distribution of the *HiTIMED* cell composition in TCGA tumors 11

Supplementary Figure 10. Cell composition differs substantially and captures sample heterogeneity using *HiTIMED*-projected proportions. Seventeen cell types were captured for each sample by tumor type 12

Supplementary Figure 11. Sensitive analysis comparing outputs from two Cox models with or without cell type proportions adjusted in kidney clear cell carcinoma 13

Supplementary Figure 12. Kaplan-Meier survival curves for *HiTIMED* cells estimates in TCGA tumors 14

Supplementary Figure 13. *HiTIMED* cell comparison and Kaplan-Meier survival curves across immune/angiogenic hot and cold 15

Supplementary Figure 14. *HiTIMED* immune and angiogenic proportions across C1-C6 subtyped TCGA tumor 16

Supplementary Figure 15. *HiTIMED* cell comparisons between drug-sensitive and -resistant metastasized colorectal cancer 17
Supplementary Figure 16. *HiTIMED* cell comparisons in triple−negative breast cancer w/without chemotherapy 18
Supplementary Figure 17. Performance comparison across iterations on CpGs selected in *HiTIMED* for immune and angiogenic cell projection. 19

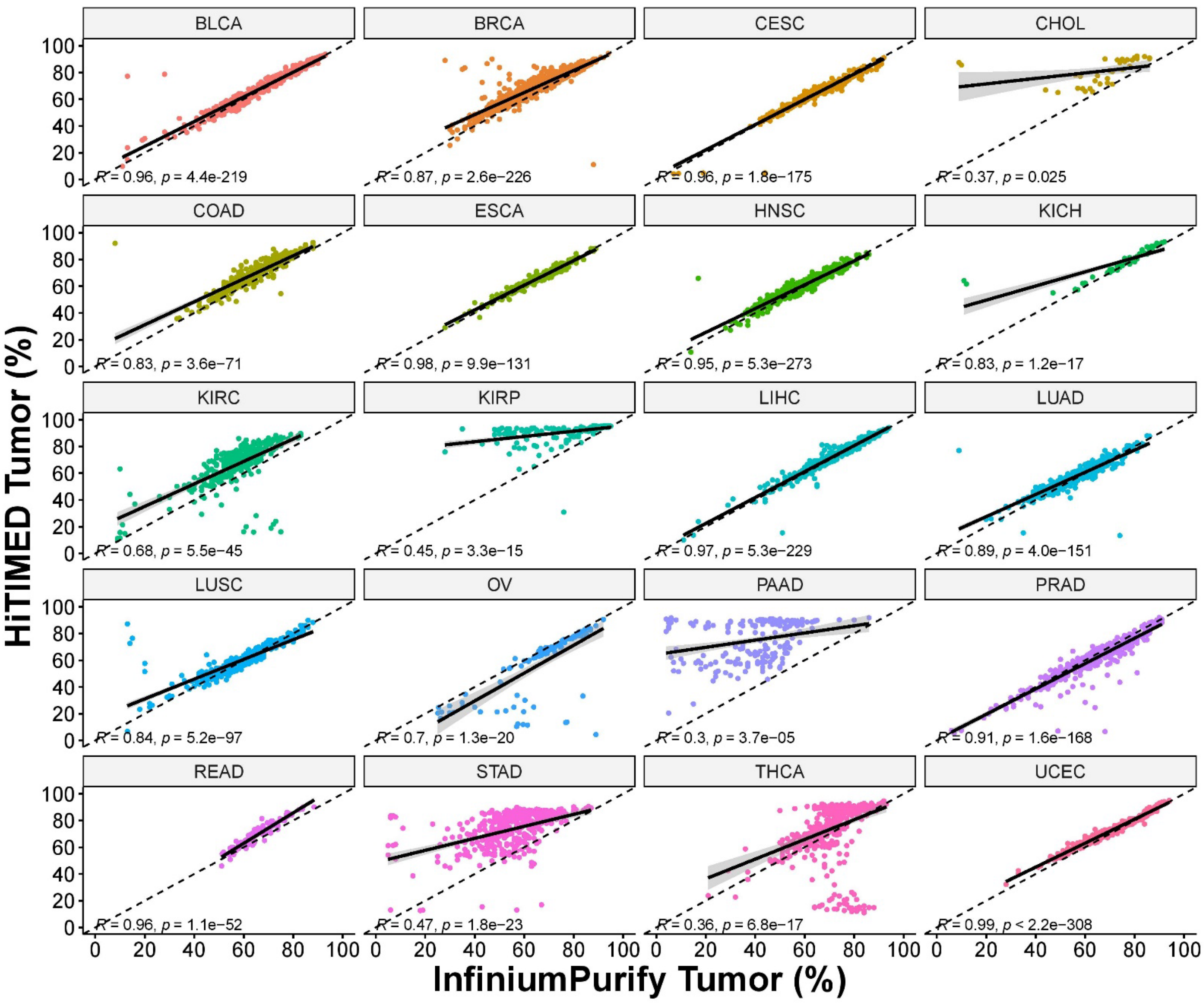

Supplementary Figure 1. Correlation between *HiTIMED* tumor and *InfiniumPurify* tumor by tumor type. *HiTIMED* projected tumor proportions are highly significantly correlated with the *InfiniumPurify* predicted tumor purities across tumor types.

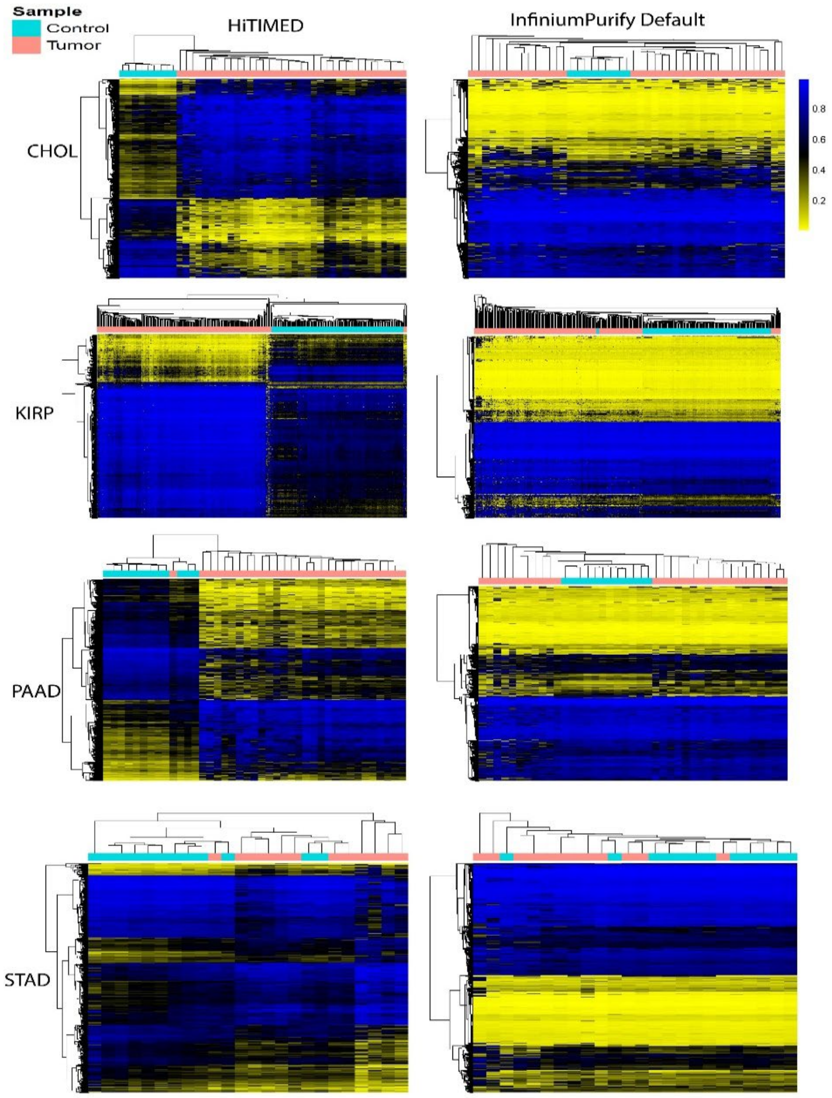

Supplementary Figure 2. Methylation state of CpGs in the *HiTIMED* tumor specific library (L1) and *InfiniumPurify* default library between tumor and normal samples across cholangiocarcinoma, kidney papillary cell carcinoma, pancreatic adenocarcinoma, and stomach adenocarcinoma.

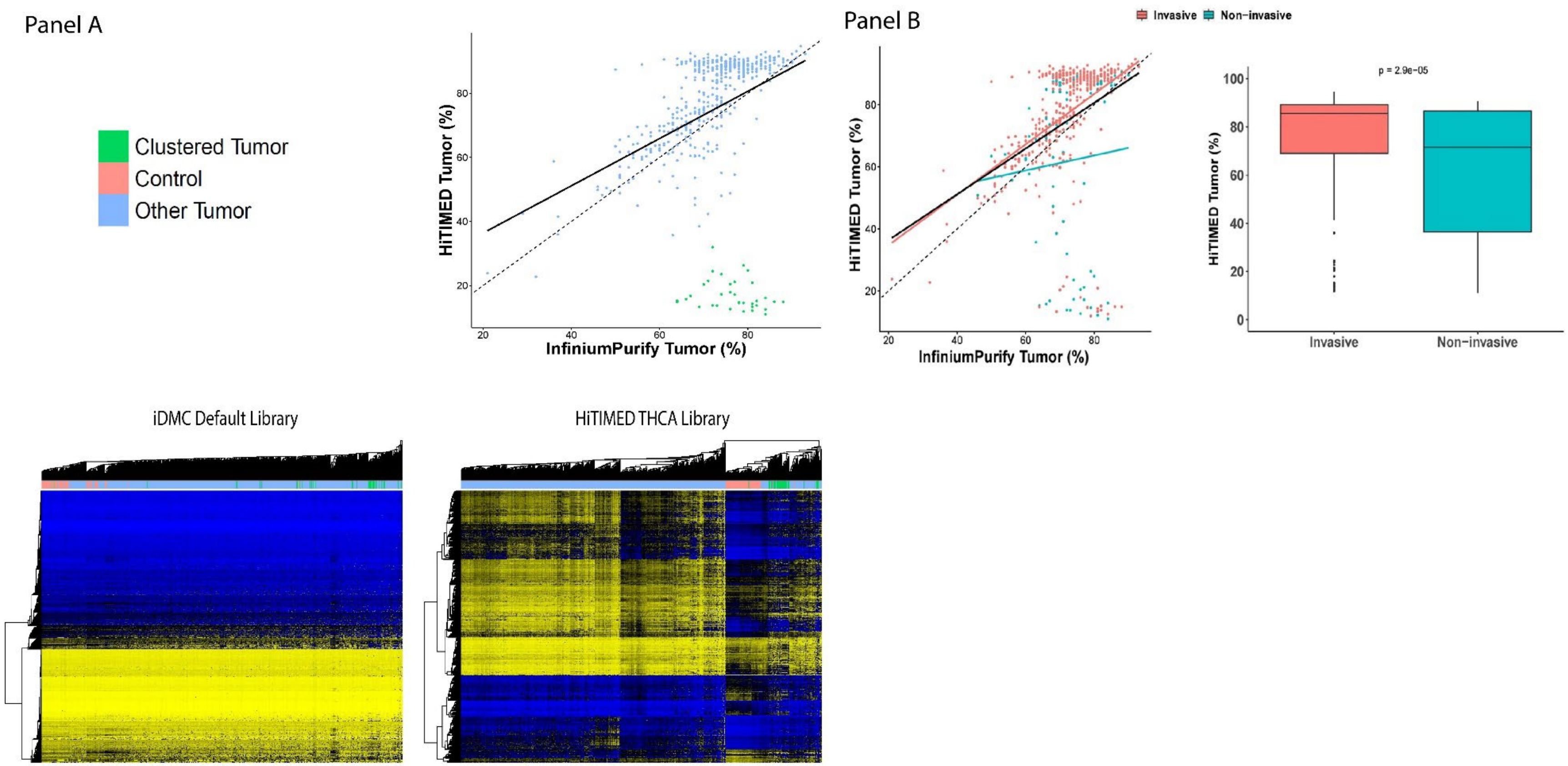

Supplementary Figure 3. *HiTIMED* tumor purity vs *InfiniumPurify* tumor purity in thyroid carcinoma. **Panel A.** A cluster of *HiTIMED*-predicted tumor purity low but *InfiniumPurify*-predicted high tumor was identified and colored in heatmaps. **Panel B.** *HiTIMED* tumor proportion in thyroid carcinoma colored by invasive and non-invasive tumor type.

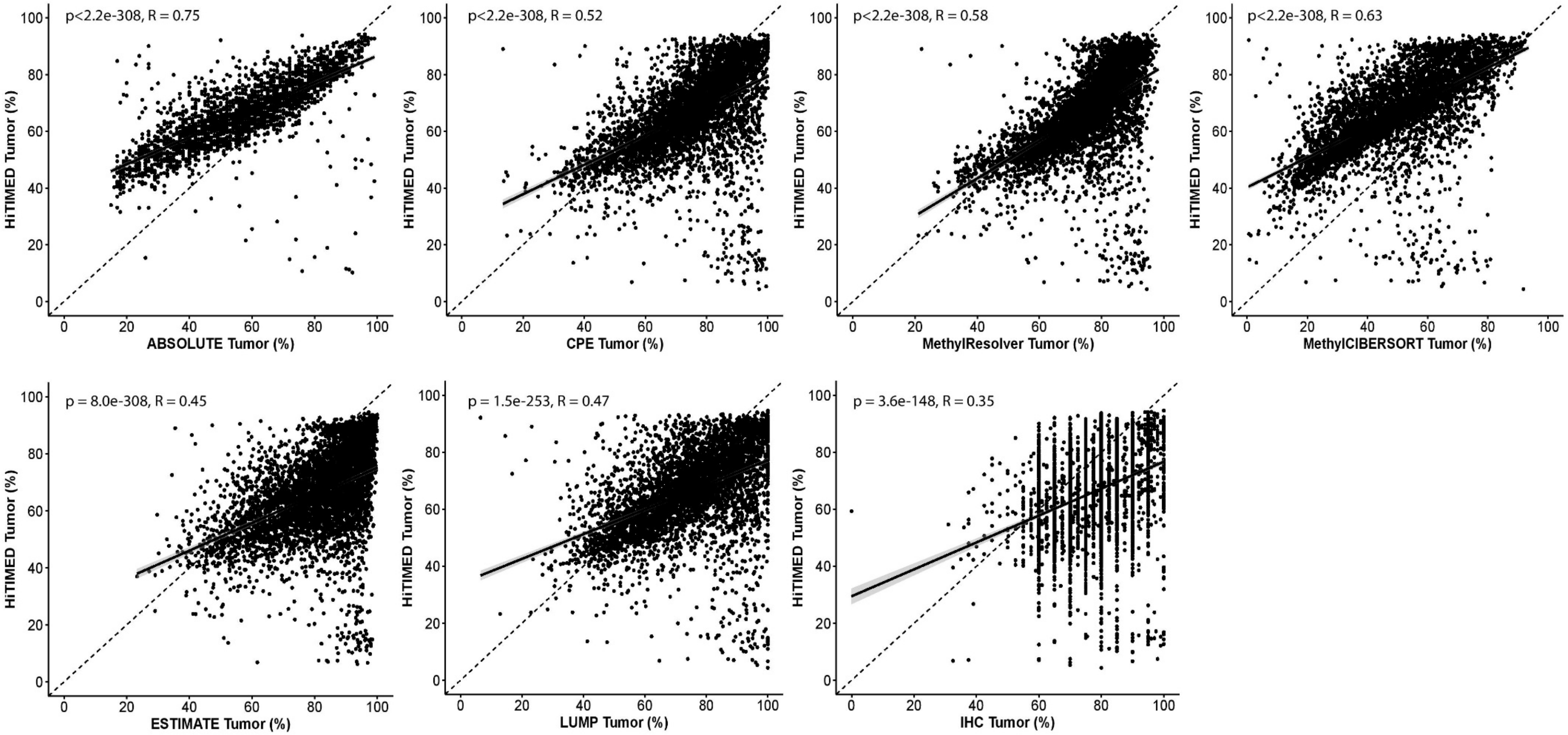

Supplementary Figure 4. *HiTIMED* tumor proportion vs other method predicted tumor proportion.

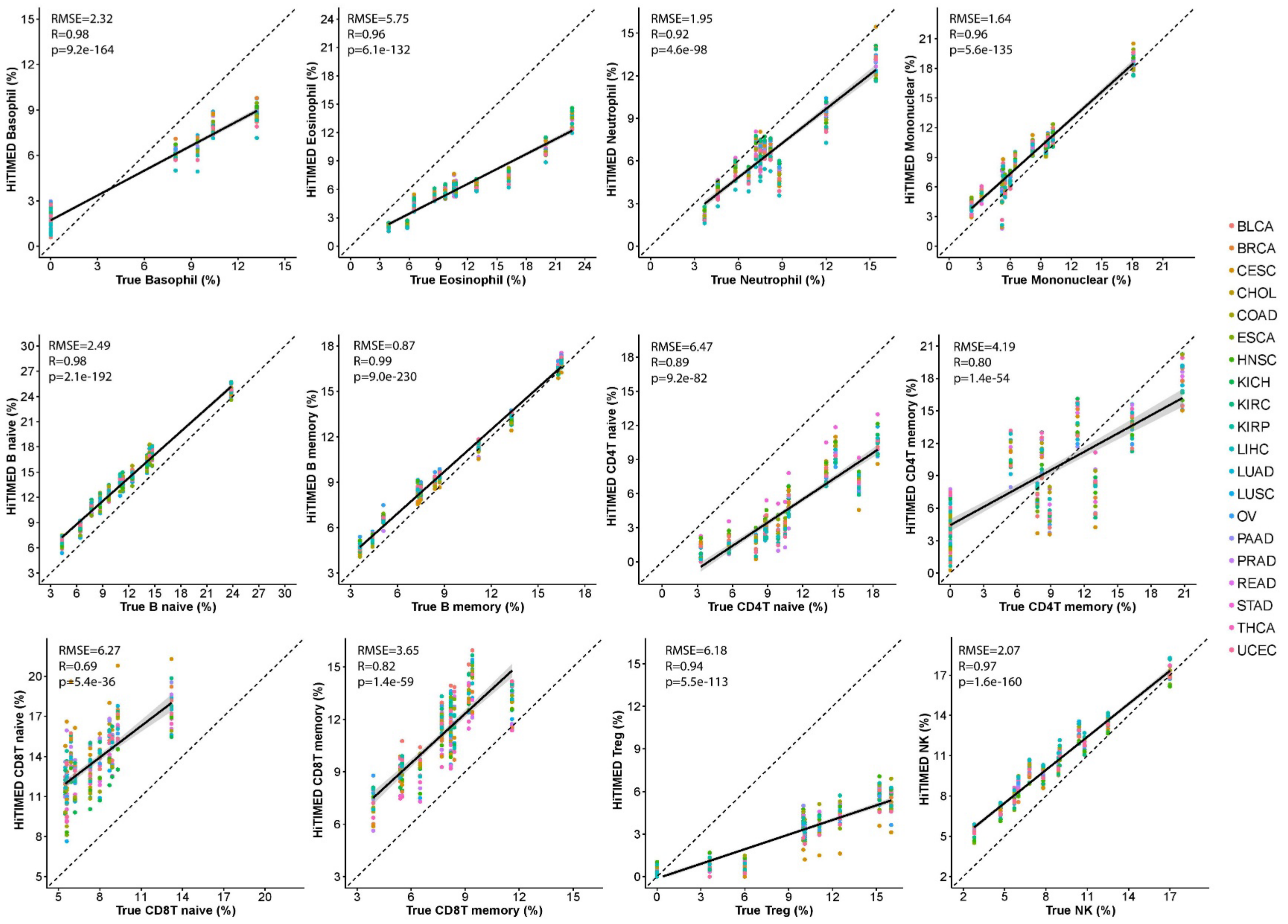

Supplementary Figure 5. *HiTIMED* immune cell proportions vs true immune cell proportions in artificial mixtures.

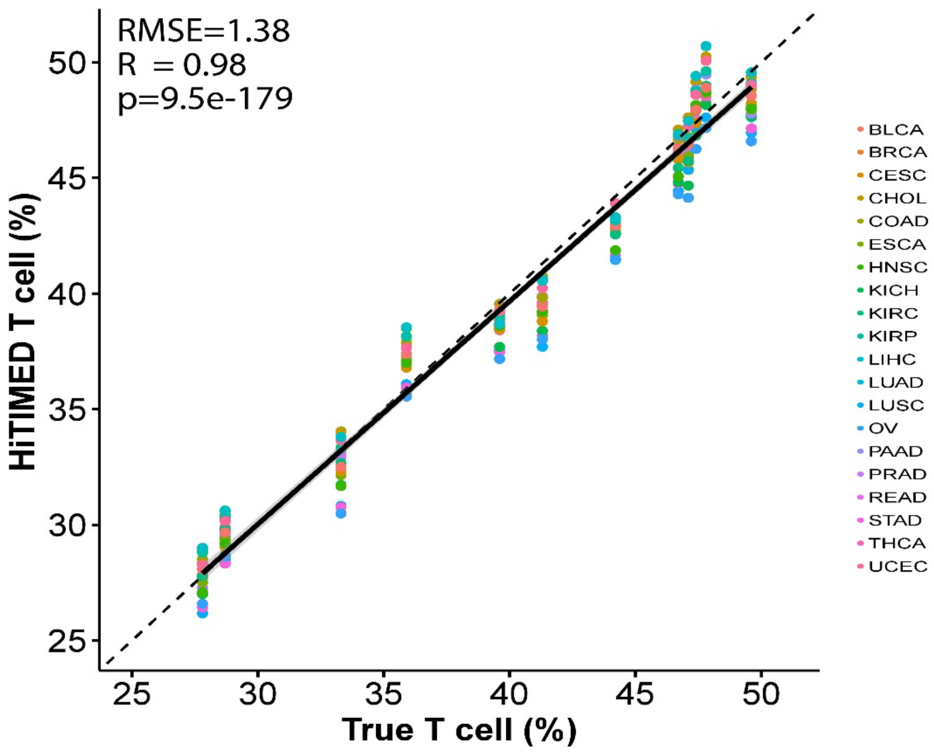

Supplementary Figure 6. *HiTIMED* T cell proportion vs true T cell proportion in artificial mixtures.

Note: T cell proportions correspond to the sum of CD4T naive and memory, CD8 naive and memory and T regulatory cells.

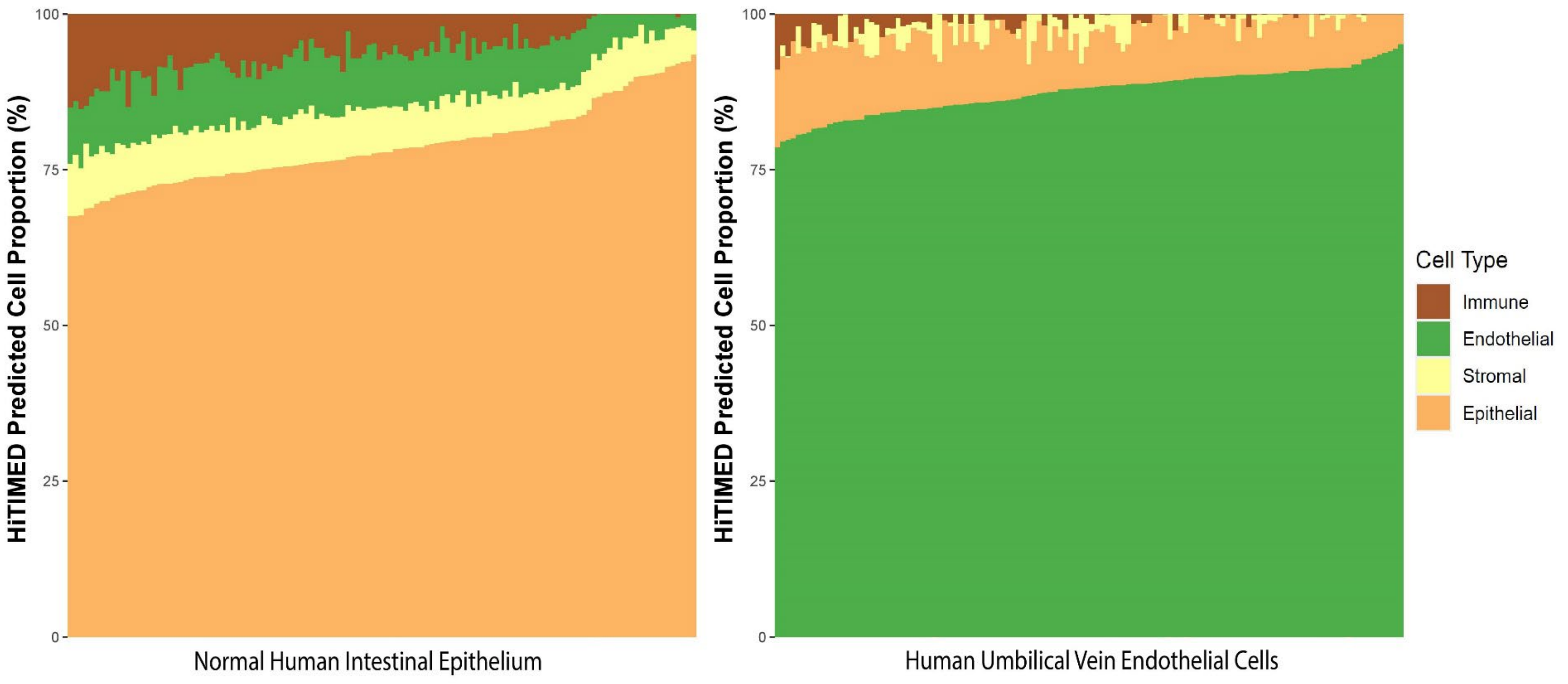

Supplementary Figure 7. *HiTIMED* cell composition in human normal intestinal epithelium and umbilical vein endothelial cells.

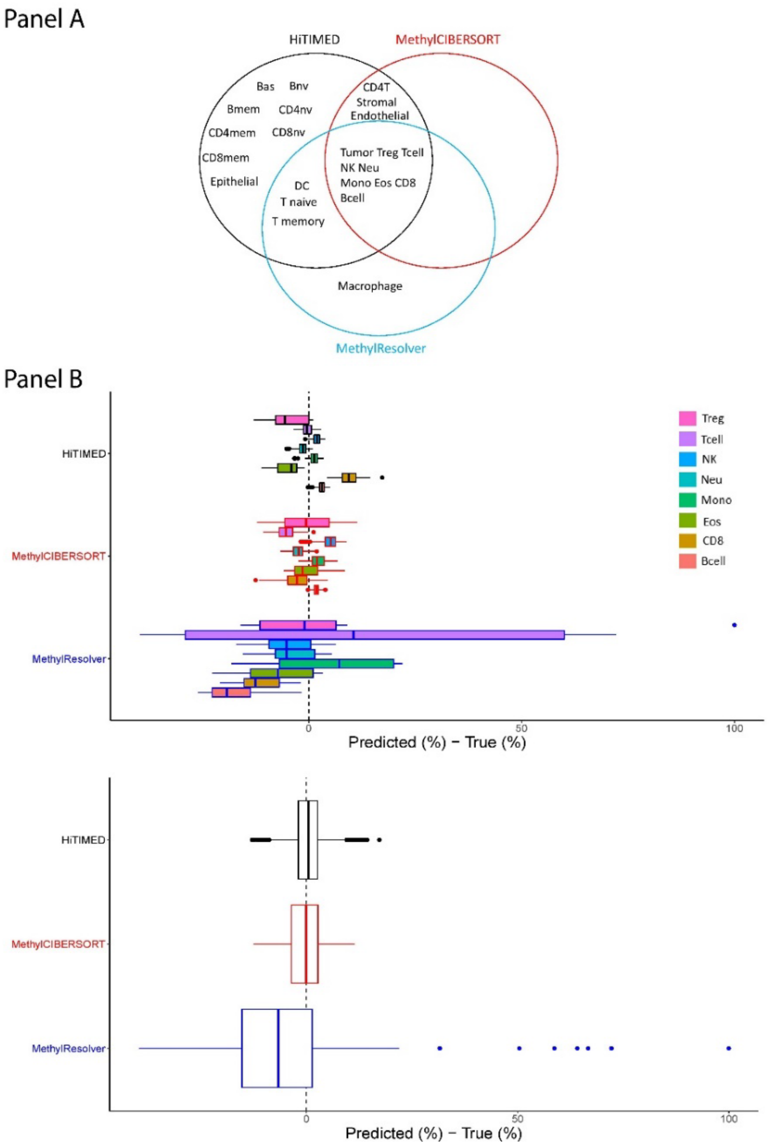

Supplementary Figure 8. Performance comparison across *HiTIMED, MethylCIBERSORT, and MethylResolver* using artificial mixtures.

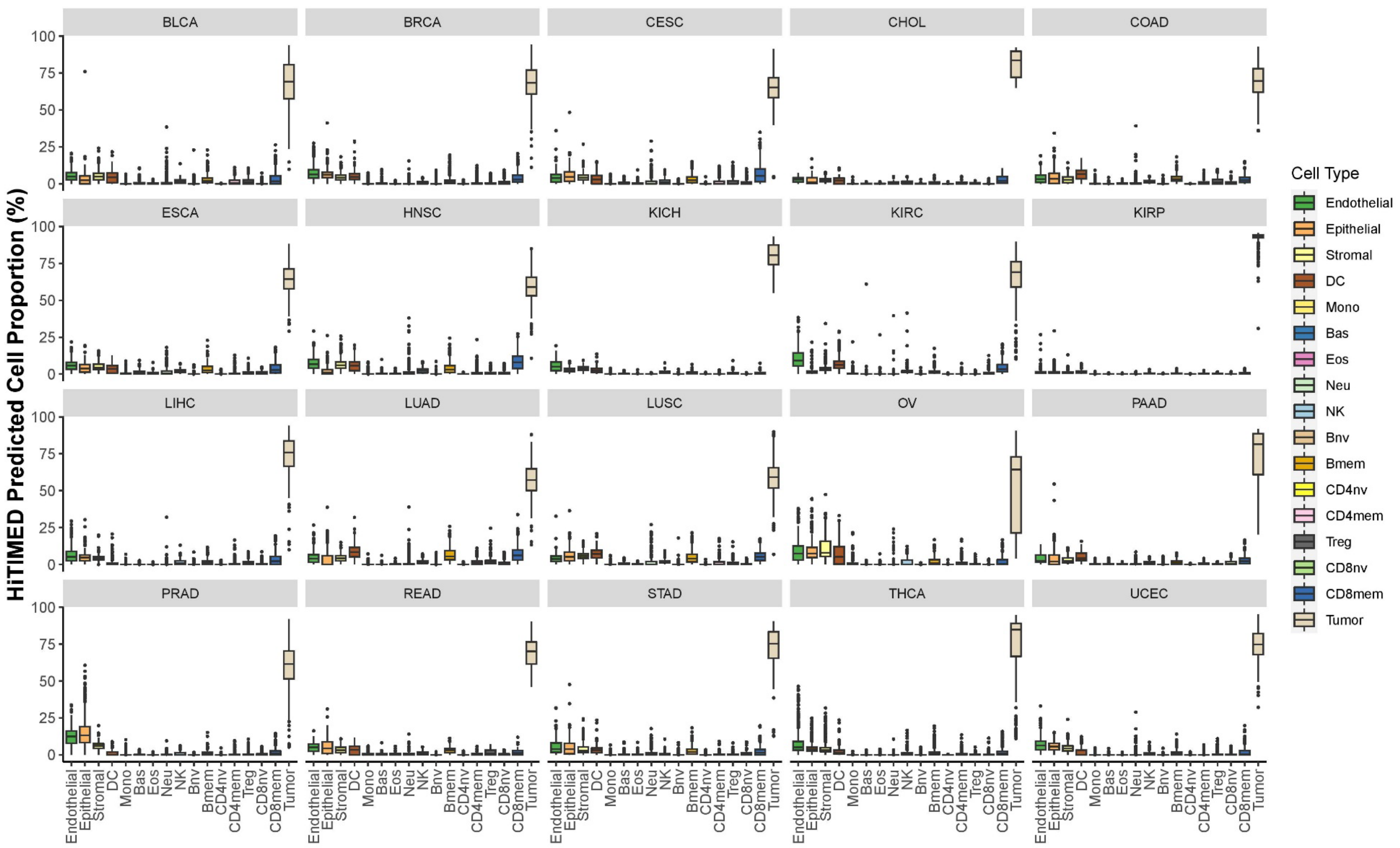

Supplementary Figure 9. The distribution of the *HiTIMED* cell composition in TCGA tumors.

Panel A

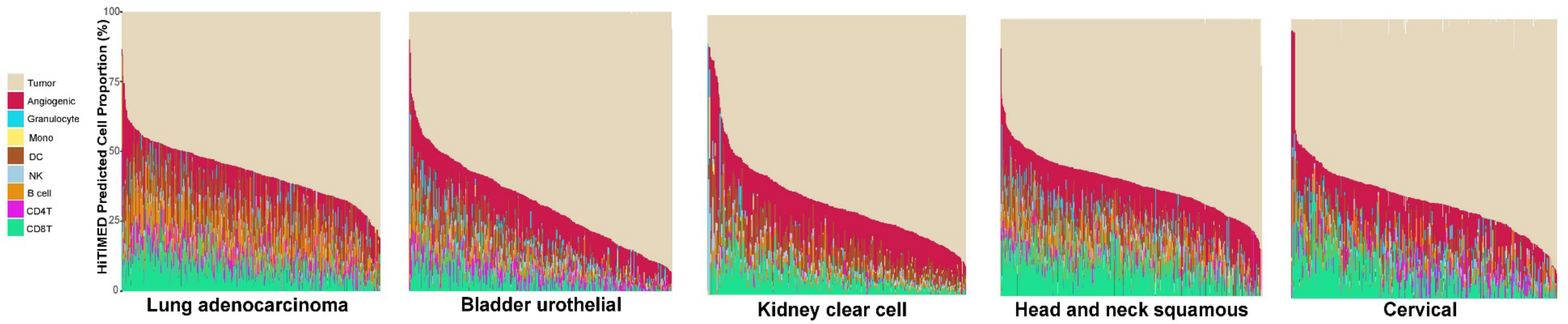

Tumor type	HiTIMED-projected immune variance
**Lung adenocarcinoma**	**156.83**
**Bladder urothelial**	**152.33**
**Kidney clear cell**	**147.83**
**Head and neck squamous**	**134.17**
**Cervical**	**118.69**
Lung squamous	96.94
Thyroid	93.45
Colon	91.15
Liver hepatocellular	91.07
Breast invasive	90.86

Panel B

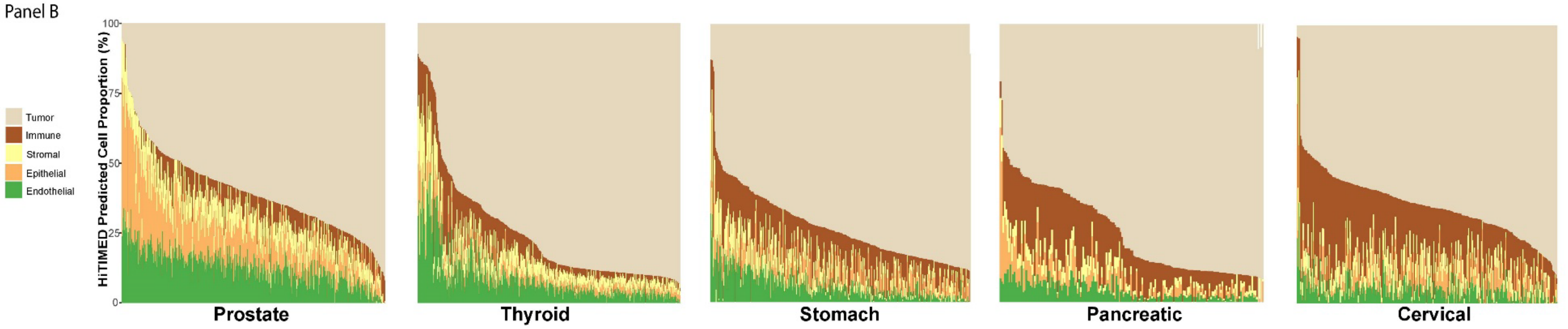

Tumor type	HiTIMED-projected angiogenic variance
**Prostate**	**284.73**
**Thyroid**	**208.77**
**Stomach**	**112.98**
**Pancreatic**	**105.84**
**Cervical**	**89.01**
Lung adenocarcinoma	88.33
Liver hepatocellular	79.43
Kidney clear cell	79.15
Breast invasive	73.19
Endometrial	66.68

Supplementary Figure 10. Cell composition differs substantially and captures sample heterogeneity using *HiTIMED*-projected proportions. Seventeen cell types were captured for each sample by tumor type.

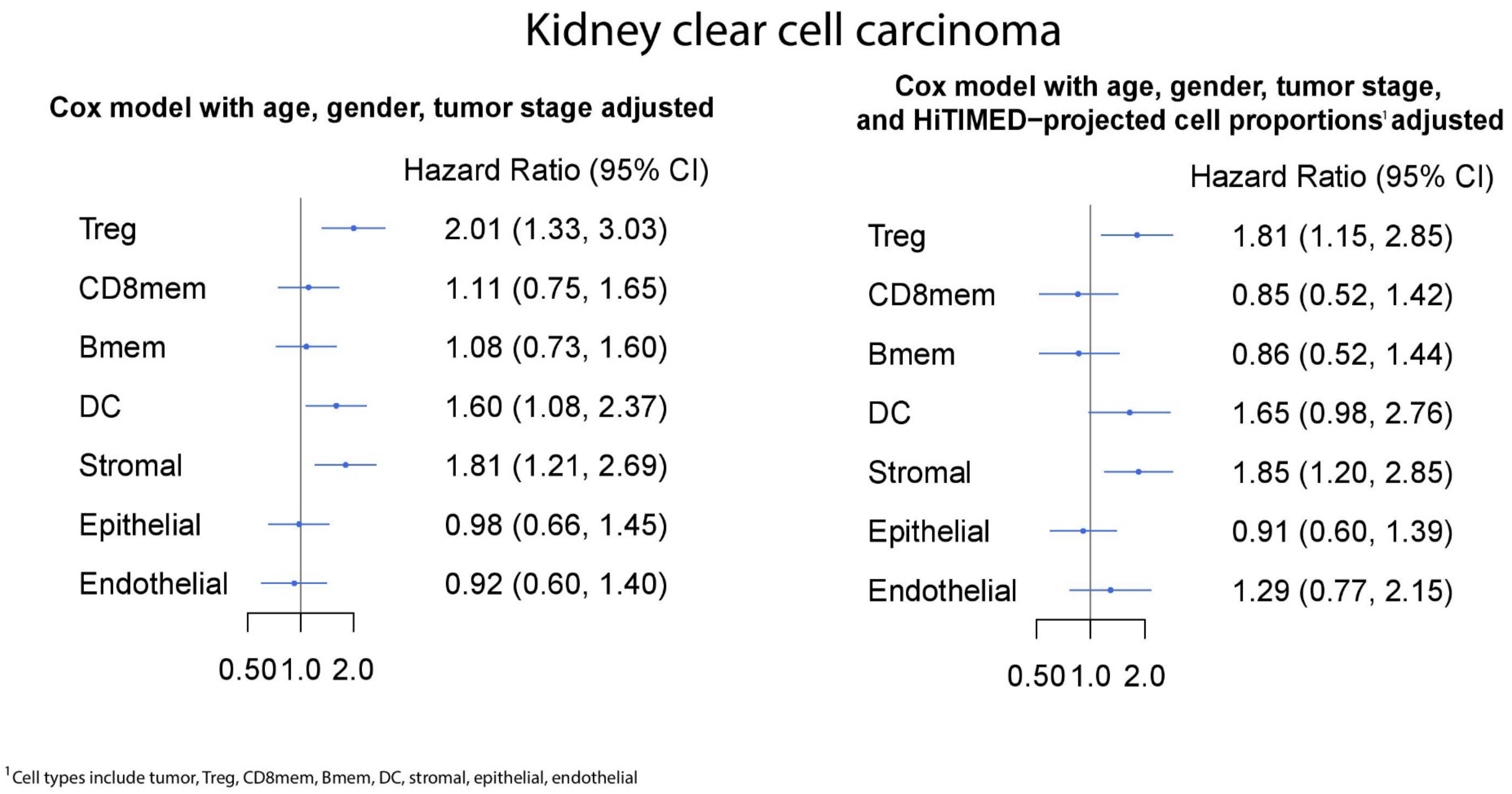

Supplementary Figure 11. Sensitive analysis comparing outputs from two Cox models with or without cell type proportions adjusted in kidney clear cell carcinoma.

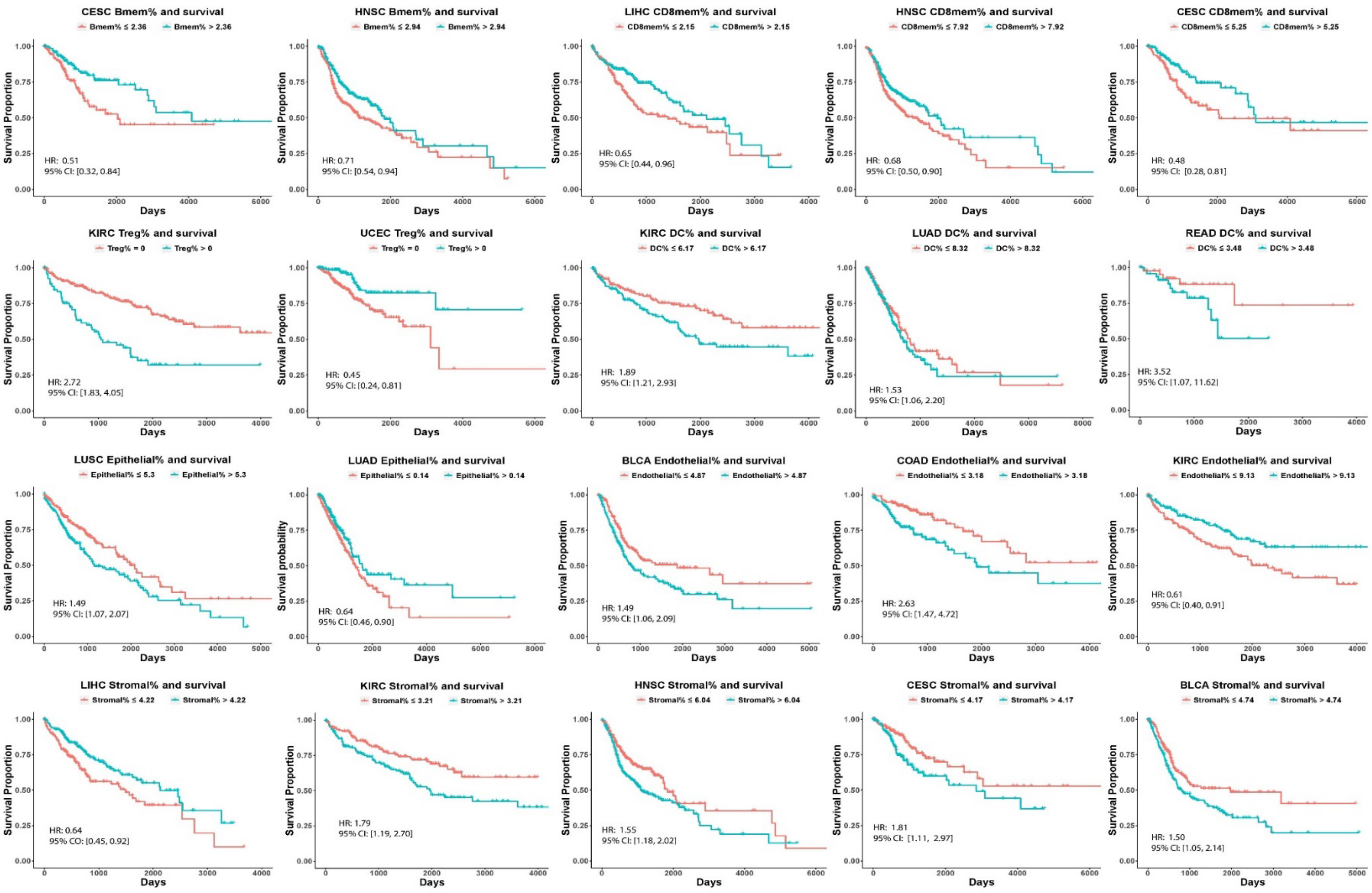

Supplementary Figure 12. Kaplan-Meier survival curves for *HiTIMED* cells estimates in TCGA tumors. Hazard ratios were calculated from the Cox proportional hazard models with age, gender, and tumor proportion adjusted (gender was not adjusted for gender-specific tumors).

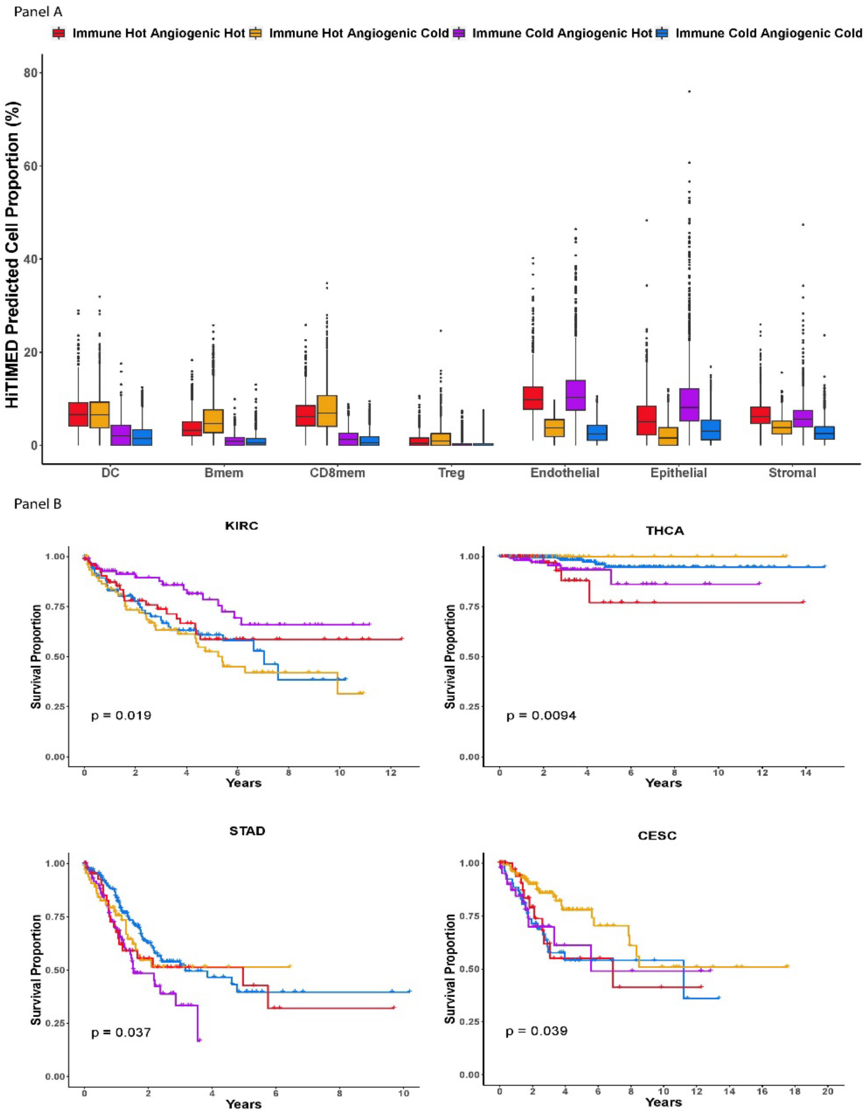

Supplementary Figure 13. Panel A. *HiTIMED* cell comparison and **Panel B.** Kaplan-Meier survival curves across immune/angiogenic hot and cold tumors. P-values were calculated from the log-rank tests.

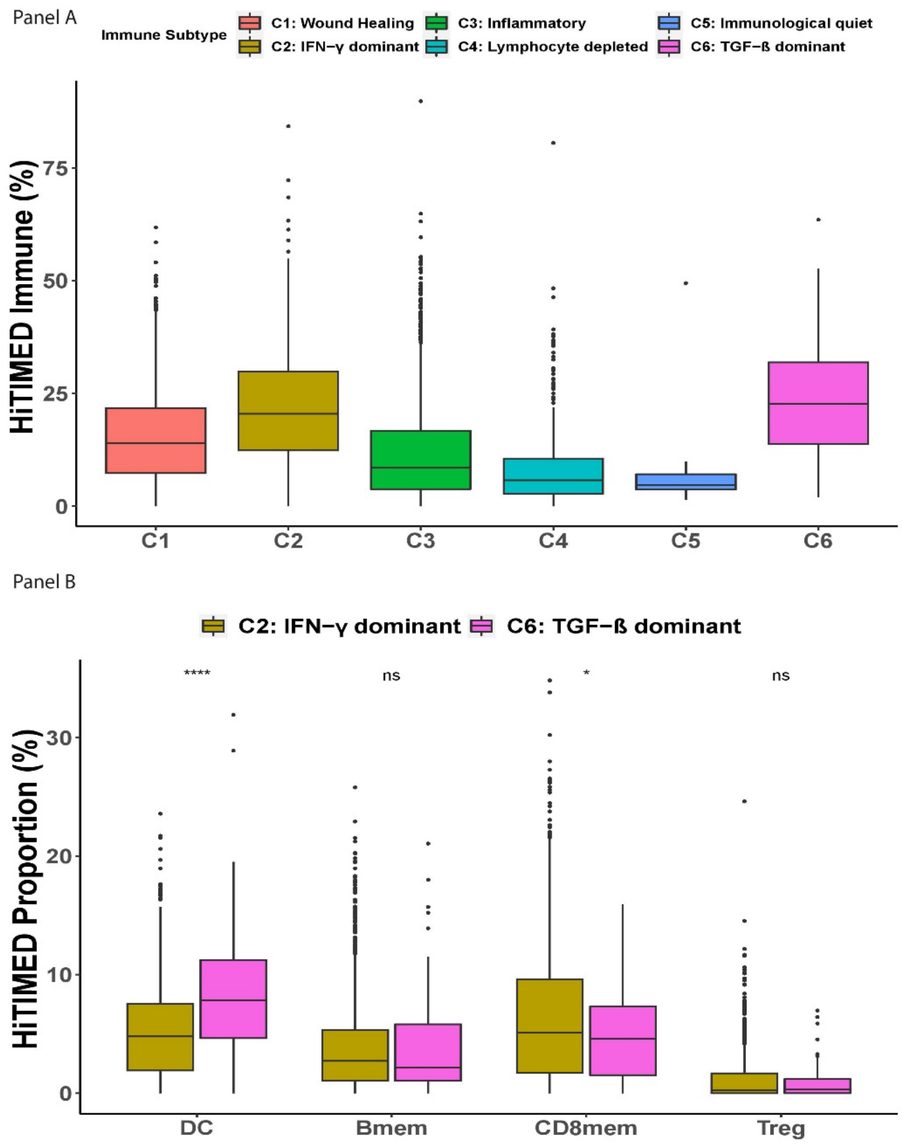

Supplementary Figure 14. *HiTIMED* immune and angiogenic proportions across C1-C6 subtyped TCGA tumor.

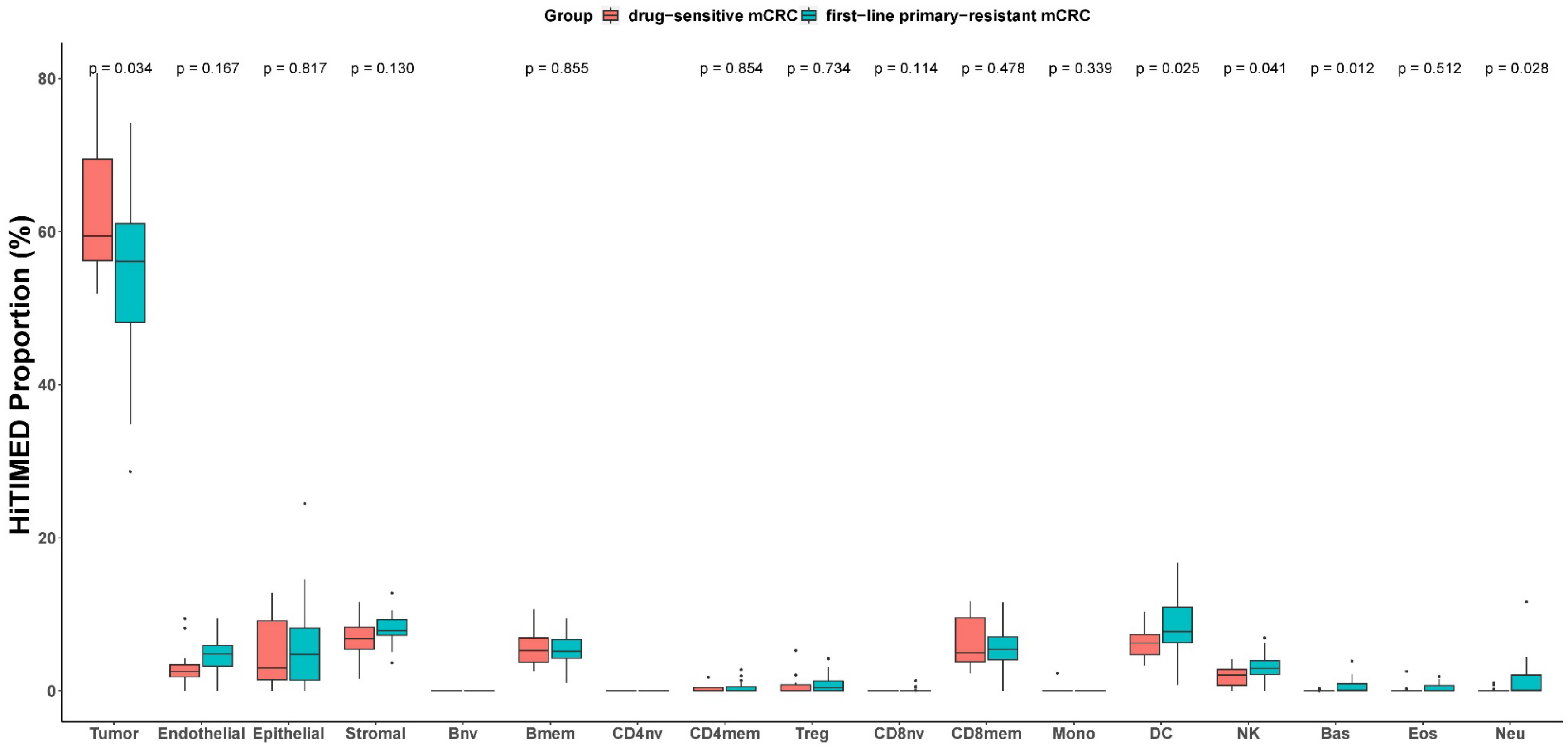

Supplementary Figure 15. *HiTIMED* cell comparisons between drug-sensitive and -resistant metastasized colorectal cancer.

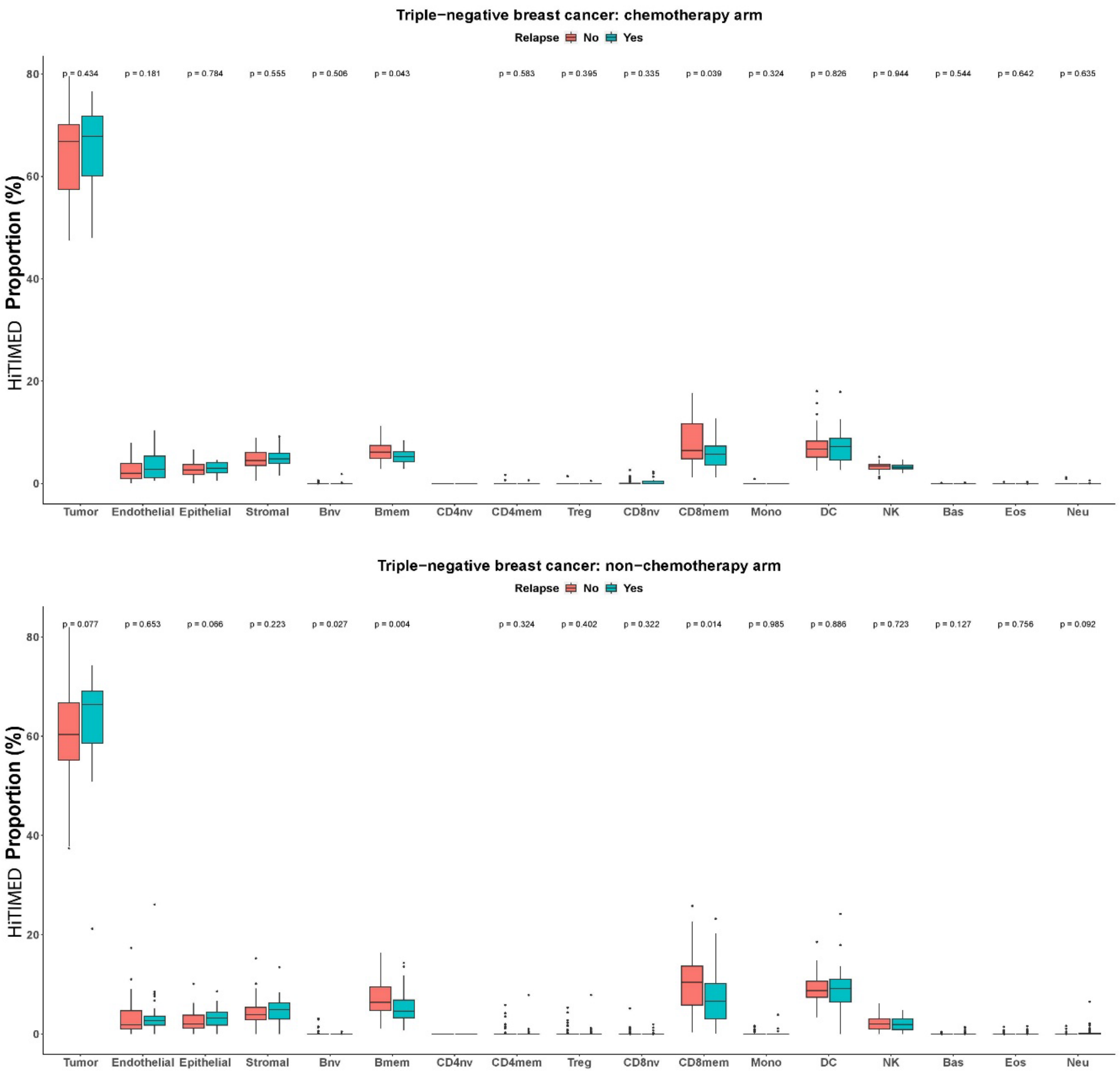

Supplementary Figure 16. *HiTIMED* cell comparisons in triple−negative breast cancer w/without chemotherapy.

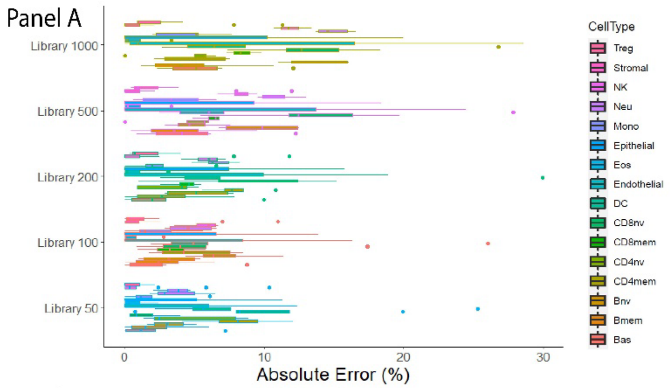

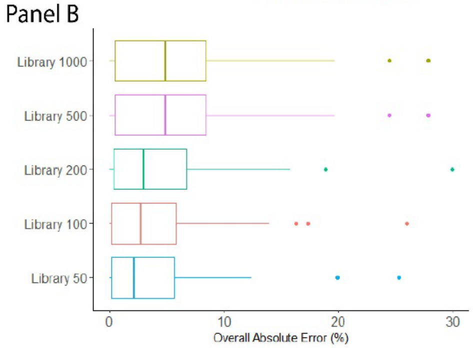

Library	min	quantile 1	median	mean	quantile 3	max
Library 50	0	0.1	2.13	3.5	5.62	25.28
Library 100	0	0.11	2.67	3.71	5.75	26
Library 200	0	0.33	2.98	4.38	6.66	29.93
Library 500	0	0.46	4.89	5.68	8.4	27.83
Library 1000	0	0.97	5.25	6.74	11.3	28.56

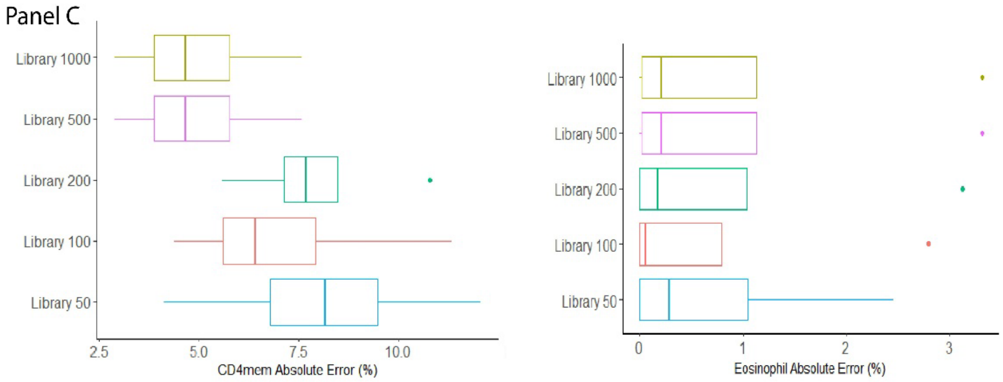

Supplementary Figure 17. Performance comparison across iterations on CpGs selected in *HiTIMED* for immune and angiogenic cell projection.
